# Idiographic Lapse Prediction With State Space Modeling: Algorithm Development and Validation Study

**DOI:** 10.2196/73265

**Published:** 2025-06-03

**Authors:** Eric Pulick, John Curtin, Yonatan Mintz

**Affiliations:** 1 Department of Industrial and Systems Engineering College of Engineering University of Wisconsin–Madison Madison, WI United States; 2 Department of Psychology College of Letters & Science University of Wisconsin–Madison Madison, WI United States

**Keywords:** mental health, digital health, alcohol use disorder, substance use disorder, mobile health, mHealth, personalized medicine, digital therapeutics

## Abstract

**Background:**

Many mental health conditions (eg, substance use or panic disorders) involve long-term patient assessment and treatment. Growing evidence suggests that the progression and presentation of these conditions may be highly individualized. Digital sensing and predictive modeling can augment scarce clinician resources to expand and personalize patient care. We discuss techniques to process patient data into risk predictions, for instance, the lapse risk for a patient with alcohol use disorder (AUD). Of particular interest are idiographic approaches that fit personalized models to each patient.

**Objective:**

This study bridges 2 active research areas in mental health: risk prediction and time-series idiographic modeling. Existing work in risk prediction has focused on machine learning (ML) classifier approaches, typically trained at the population level. In contrast, psychological explanatory modeling has relied on idiographic time-series techniques. We propose state space modeling, an idiographic time-series modeling framework, as an alternative to ML classifiers for patient risk prediction.

**Methods:**

We used a 3-month observational study of participants (N=148) in early recovery from AUD. Using once-daily ecological momentary assessment (EMA), we trained idiographic state space models (SSMs) and compared their predictive performance to logistic regression and gradient-boosted ML classifiers. Performance was evaluated using the area under the receiver operating characteristic curve (AUROC) for 3 prediction tasks: same-day lapse, lapse within 3 days, and lapse within 7 days. To mimic real-world use, we evaluated changes in AUROC when models were given access to increasing amounts of a participant’s EMA data (15, 30, 45, 60, and 75 days). We used Bayesian hierarchical modeling to compare SSMs to the benchmark ML techniques, specifically analyzing posterior estimates of mean model AUROC.

**Results:**

Posterior estimates strongly suggested that SSMs had the best mean AUROC performance in all 3 prediction tasks with ≥30 days of participant EMA data. With 15 days of data, results varied by task. Median posterior probabilities that SSMs had the best performance with ≥30 days of participant data for same-day lapse, lapse within 3 days, and lapse within 7 days were 0.997 (IQR 0.877-0.999), 0.999 (IQR 0.992-0.999), and 0.998 (IQR 0.955-0.999), respectively. With 15 days of data, these median posterior probabilities were 0.732, <0.001, and <0.001, respectively.

**Conclusions:**

The study findings suggest that SSMs may be a compelling alternative to traditional ML approaches for risk prediction. SSMs support idiographic model fitting, even for rare outcomes, and can offer better predictive performance than existing ML approaches. Further, SSMs estimate a model for a patient’s time-series behavior, making them ideal for stepping beyond risk prediction to frameworks for optimal treatment selection (eg, administered using a digital therapeutic platform). Although AUD was used as a case study, this SSM framework can be readily applied to risk prediction tasks for other mental health conditions.

## Introduction

Data-driven psychological modeling methods can help researchers leverage increasingly available patient data to better understand and treat pressing mental health conditions [1-3]. In parallel, digital interventions introduce new, low-burden treatment pathways to patients [4,5]. Together, these advancements give providers exciting new tools for low-barrier, personalized risk monitoring and treatment, which are core elements of continuing care for chronic mental health conditions [6]. Continuing care is an important recovery support for chronic conditions, such as alcohol use disorder (AUD) and other substance use disorders (SUDs) [7,8], but is underavailable and underutilized [9]. Novel approaches are needed to close this gap.

Advances in personal sensing have spurred new modeling approaches by empowering researchers to collect rich longitudinal patient datasets [10]. Ubiquitous mobile devices, such as smartphones and wearables, increase both the types and quantities of available measurements. Diary-type methods, such as ecological momentary assessment (EMA) [11], have long been an important way to gather *in situ* measurements of a patient’s experience or mental state, and this type of active data collection has been made easier by the prevalence of mobile devices [12]. In addition to actively queried measurements like EMA, researchers can also use mobile devices to gather patient data passively, such as physiological measurements or a patient’s GPS location. The explosion of data availability has led researchers to examine mental health conditions in more quantitative ways through 2 (often parallel) perspectives: explanation-focused modeling and prediction-focused modeling.

Explanation-focused modeling has largely turned to time-series methods to better understand the dynamics of different mental health conditions [13], sometimes explicitly focused on identifying causal mechanisms [14]. This line of research has generated a rich set of modeling approaches, typically using various forms of network analysis or structural equation modeling [15-23]. These approaches create mathematical models that explain variations in individual patient behavior over time, often implemented as multilevel models that separate group-level and person-specific model effects. Implicit in this separation is the notion of an idiographic-nomothetic spectrum [24-27]. While some behaviors may be well-described by nomothetic principles that hold *on average* across a population, there is no guarantee that those principles will apply well to a particular individual. Importantly, there is evidence suggesting that the presentation and progression of many mental health conditions are meaningfully individualized (eg, in mood dynamics [28,29], suicidal thoughts [30], depression [31], and panic disorder [32]). These examples emphasize the importance of personalized modeling approaches that can capture person-specific heterogeneity. Note that here we use “idiographic” synonymously with “personalized” and “person-specific,” describing when separate models are fit to each individual, even if group-level information is incorporated into the patient’s model in some way. Under a strict definition of idiographic, such a model might be better described as “idiothetic” [33]. For our purposes, the important distinction is whether each participant has a separate model.

In contrast to explanatory modeling, prediction-focused modeling emphasizes building models that predict behavior well, even if doing so does not necessarily give insights into the underlying behavior-generating process [34]. For instance, many mental health conditions exhibit high-acuity binary outcomes (eg, lapse in individuals with SUDs, suicidal ideation, panic attacks, binge eating, etc) that fit naturally into a machine learning (ML) prediction framework. ML classifier methods like logistic regression (LR) or gradient boosting models have used digital sensing data to successfully predict, for example, patient risk for AUD lapse or binge eating [6,35-38]. This type of risk assessment is an important component of successful patient intervention. In situations where treatments may be costly or scarce, such as appointments with clinicians, accurate patient risk assessment could help direct treatment resources to high-risk individuals. In settings like digital therapeutics, where intervention costs and resource scarcity are less relevant, accurate risk assessment could inform the urgency, intensity, or type of a just-in-time adaptive intervention [4].

However, ML classification approaches have 2 important drawbacks with respect to idiographic risk prediction. First, it is difficult to fit classifier models idiographically when outcomes are rare, so they are often fit at the population level instead. In the most extreme case, an exclusively person-specific model cannot be fit at all until the positive outcome label (eg, lapse) is observed. In practice, a person-specific classifier model might not show adequate performance until it can train on a sufficient number of positive outcomes, at which point it may no longer be relevant (eg, a person with SUD would likely have already relapsed). These concerns can sometimes be alleviated by modifications to incorporate prior or group-level information, such as Bayesian estimation or multilevel modeling, depending on the classifier approach being used.

The second drawback of classifier methods, however, is entirely structural: they are not time-series methods. Classifier models are trained only to make a prediction for a particular outcome, such as the probability of a patient lapsing today, and thus, they do not give insights into how a patient’s behavior will evolve over time. If a clinician is also interested in a patient’s lapse risk for other time windows (eg, tomorrow or within the next week), a separate classifier model must be trained for every such circumstance. While this type of predictive capability is clearly important, our broader goal is to use quantitative modeling to inform personalized treatment recommendations. This involves understanding “*what* treatment, by *whom*, is most effective for *this* individual with *that* specific problem under *which* set of circumstances” [39]. Models that output a single predictive risk score provide limited insights into these questions. A dynamic model describing how relevant aspects of a patient’s state of mind evolve over time would be much more helpful. Time-series models directly capture these behavioral dynamics as part of model fitting *and* can be used to produce risk scores (eg, by simulating trajectories of patient behavior, including responses to different treatment approaches). Time-series models are thus better integrated into intervention planning frameworks to optimize long-run patient outcomes.

We advocate combining these explanation- and prediction-focused research areas by using idiographic, time-series models for prediction. Specifically, we propose the use of an approach called state space modeling that supports fitting person-specific time-series models of patient behavior. State space models (SSMs) originate from the field of control engineering and share much in common with structural equation modeling and network analysis. We believe that SSMs are particularly well-suited for idiographic modeling as they focus specifically on modeling the behavior of a single subject (for a deeper discussion of commonalities and differences between structural equation models and SSMs, see [40]).

To demonstrate how SSMs can be applied to personalized mental health prediction, we use the setting of predicting lapse for individuals with AUD as a case study. We provide an overview of state space modeling and present a framework for fitting person-specific SSMs for AUD lapse prediction with longitudinal EMA observations. We compare the predictive performance of SSMs to that of commonly used ML classifiers and demonstrate that this idiographic, time-series approach can have comparable or better predictive performance than ML approaches used in the literature. As part of this analysis, we explicitly characterize how predictive performance changes when more data are available for each study participant. This is an important practical consideration for the real-world use of personalized models. Further, we discuss how the SSMs we propose are readily integrated into advanced personalized intervention frameworks. Lastly, we discuss how the SSM frameworks presented here can be similarly applied to other mental health conditions beyond AUD, such as anxiety and panic disorders, depression, and suicidal ideation.

## Methods

### Ethical Considerations

This study conducted analyses to address the second aim of a larger grant-funded parent project (R01 AA024391). This parent effort collected the study data between 2017 and 2019. The University of Wisconsin-Madison Institutional Review Board approved all procedures (IRB #2015-0780). All study participants provided written informed consent prior to their participation. Study consent documentation is provided in a persistent repository (see [41]) on Open Science Framework (OSF). To protect participant data privacy, the parent study that collected these data received a Certificate of Confidentiality from the National Institutes of Health. Further, no identification of individual data is possible from this manuscript or supplementary materials. We do not present individual patient data; all data are presented for the complete population using a deidentified version of the original dataset. Participants were compensated up to approximately US $800, depending on measure adherence (see Section S2 in Multimedia Appendix 1).

### Transparency and Openness

We adhere to research transparency principles that are crucial for robust and replicable science. Data, analysis scripts, annotated results, measures, preregistration of the hypotheses and analysis plan (see [42]), and other study materials are publicly available at our OSF repository (see [41]). We report how the sample size, all data exclusions, all manipulations or procedures, and all study measures were determined. Measures and procedures that were collected as part of the parent project but were not relevant to this study have been described in [6,43]. We also provide a transparency report [44] in Multimedia Appendix 2 and a biomedical ML checklist [45] in Multimedia Appendix 3.

### Recruitment

We analyzed 148 study participants in early remission from AUD (1-8 weeks of abstinence). Participants were recruited from Madison, Wisconsin and surrounding communities through print and targeted digital advertisements and partnerships with treatment centers. The requirements for inclusion are provided in Textbox 1. For additional information regarding recruitment, please refer to the study design documentation available on OSF (see [41]) and in the parent study [6].

**Criteria for inclusion in the parent project.**

**Inclusion criteria**

1. Age ≥18 years
2. Ability to write and read English
3. Presence of at least moderate alcohol use disorder (≥4 symptoms from the Diagnostic and Statistical Manual of Mental Disorders, Fifth Edition, measured with a self-report survey during a screening visit)
4. Abstinence from alcohol for at least 1 week but no longer than 2 months
5. Willingness to use a single smartphone (their personal phone or one provided to them) while enrolled in the study

Alcohol abstinence was used as a behavioral indicator of a commitment to recovery. Although recovery may be possible without complete abstinence, clinicians typically recommend abstinence for patients who present with moderate or more severe AUD. Individuals were excluded if they exhibited severe symptoms of psychosis or paranoia, defined as scores greater than 2.2 or 2.8, respectively, on the psychosis or paranoia scales in the Symptom Checklist-90 [46]. The sample size for the parent project was determined based on power analyses for its broader set of aims. The authors of the parent project determined preliminary eligibility by phone and completed screening and obtained written informed consent at a subsequent in-person screening visit. The study period for each participant lasted up to 3 months, beginning with an instructional intake visit, which was followed by in-person visits every 30 days.

### Measures

The study collected baseline demographics and participant characteristics relevant to alcohol use at the screening and intake visits. Participants completed brief EMAs (7-10 questions) 4 times each day following prompts sent by text messages. These text messages included a link to a Qualtrics survey that was optimized for completion on their smartphones. All EMAs contained a common set of 7 questions asking about any alcohol use that had not yet been reported, current affective state (pleasantness and arousal), greatest urge to drink alcohol since the last EMA, any pleasant or positive events and any hassles or stressful events that occurred since the last EMA, and any exposure to risky situations (ie, people, places, or things) since the last EMA. The first EMA each day (the “morning EMA”) asked 3 additional questions about how likely participants were to encounter a risky situation, encounter a stressful event, and drink alcohol in the upcoming week. A copy of the morning EMA is available on OSF [41].

Although the parent study collected up to 4 EMAs per participant each day over the 3-month study period, we believe that 1 EMA per day represents a more realistic sensing burden for real-world implementation, especially for situations where patients might be followed for longer time periods [43,47]. Accordingly, we chose to compare models built using only 1 EMA response from participants each day, specifically the morning EMA. We chose the morning EMA as it contained the most informative set of questions and occurred at a similar time near the beginning of each participant’s day. The first EMA question was used to report past alcohol consumption, leaving 9 Likert-style questions for analysis. Note that all 4 daily EMAs were used, along with in-person follow-up visits, to collect self-reported lapse (ie, outcome label) information, but only the Likert-style questions from the morning EMA were used as inputs in our modeling. We do not believe this has a practical impact on our findings, as a future implementation could equivalently collect the same lapse information using only 1 EMA per day. The original study [6] had 151 participants, but we excluded 3 participants who responded to fewer than 25% of the morning EMAs during their study period, leaving 148 participants for analysis.

### Labels and Prediction Tasks

For each participant, we constructed binary labels indicating if the participant drank alcohol each day. Days began and ended at 4 AM. If the participant self-reported alcohol use during this interval, the day was labeled as 1; otherwise, it was labeled as 0. As all study participants stated a goal of abstinence, any use of alcohol was inconsistent with their goals. For brevity, we refer to any such goal-inconsistent use as a lapse. We established the 4 AM start and end time for each day to align better with the waking hours between periods of sleep. For instance, if a participant’s study began on January 1 and they experienced a lapse at 2 AM on January 2, this lapse would be included as part of day 1 in their study. For 2 participants who typically woke up before 4 AM, study days were defined to begin and end at 3:30 AM.

To provide a richer view of how well each method assesses participant risk, we compared the predictive performance of the methods on 3 lapse prediction tasks ranging from same-day to week-long windows, as given in Textbox 2. Window-style predictions were only generated for days where the window was contained entirely within the participant’s study period (ie, for a participant with a 90-day study, 7-day window predictions were only made up to day 83).

**Prediction tasks.**

Given a participant’s ecological momentary assessment data from day 1 to day *t* and lapse data from day 1 to day *t*–1, our models made lapse predictions for 3 intervals of increasing length:

1. Predict the risk of lapse on day *t* (same-day lapse prediction)

2. Predict the risk of lapse occurring on any day between day *t* and day *t+*3, inclusive (lapse within 3 days)

3. Predict the risk of lapse occurring on any day between day *t* and day *t+*7, inclusive (lapse within 7 days)

### State Space Modeling

SSMs are equations that describe how a system changes over time. This framework comes from the field of control engineering and was critical, for instance, in the navigation and control of rockets in the Apollo space program [48,49]. Since then, the same modeling framework has proven valuable in many domains, such as ecology (eg, modeling wildlife population dynamics [50]) and psychology (eg, modeling affective mood dynamics [51]). State space modeling has many commonalities with structural equation modeling [40], often used for explanatory psychological modeling, but typically focuses on modeling the dynamics of a single subject.

The “state” of a system is something that usually cannot be observed directly. For a rocket, this might be its exact position or speed. In ecology, it could refer to the true size of an animal population. For more abstract systems, such as those in psychology, it might describe the intensity of a particular emotion or some other mental construct. The core insight of SSMs is the separation of a process model from an observation model. The process model governs how that system state changes over time, while the observation model describes how we can gather, often noisy, measurements of the system state. For a physical system like a rocket, the process model would be the relevant laws of physics, while the observation model would come from the noisy sensors we use to measure the system. For psychological systems, the correct, causal process model may not be known, and discovering or defining such a model is not the focus of this study. Instead, we seek to demonstrate that simple models can be good approximations for complex psychological processes and can thus be useful for patient risk assessment.

In our state space modeling, the unobserved state (also called the hidden or latent state) can be thought of as representing a set of mental constructs, relevant to lapse, that evolve from one day to the next. We cannot observe these constructs directly and instead rely on passively or actively sensed data from the participants. In this study, we observed the participants’ responses to 9 EMA questions each morning and their lapse behavior each day. For each study participant, we defined 2 key equations: the observation equation and the transition (or process) equation.

**Figure 1:**
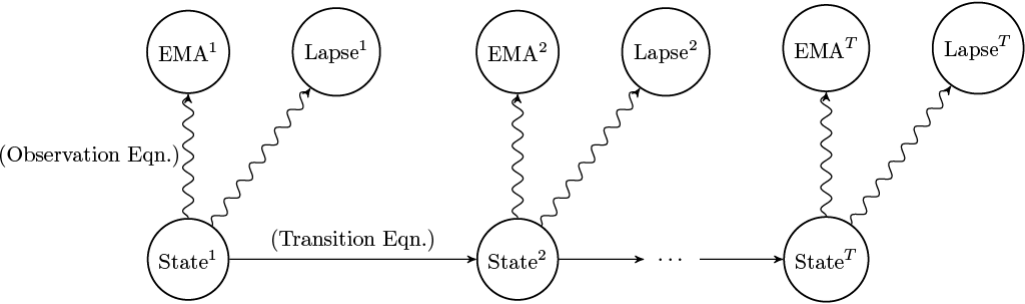
Visual representation of state space modeling for this alcohol use disorder study, following the time series for a single participant from day 1 to the end of their study period (day *T*). This modeling framework explains observable quantities (ie, a participant’s lapse decision and ecological momentary assessment [EMA] responses each day) in terms of hidden states (ie, the participant’s state of mind) that change over time. The observation equation explains how the participant’s hidden state produces EMA responses and lapses each day, while the transition equation explains how the hidden state evolves from one day to the next.

The observation equation mathematically defines the relationship between the quantities we cannot observe (the participant’s mental states) and those that we can observe (how they respond to EMAs and whether they lapse). The transition equation describes how the hidden state evolves over time. In our modeling, time steps are discretized as days of the study period. Figure 1 provides a visualization of the modeling framework. Note that this representation is a more general formulation of other autoregressive models, such as vector autoregression, that model observed quantities as functions of previously observed quantities. SSMs readily handle missing data without the need for deletion or imputation, as measured quantities are used for inference about the hidden state and not directly to predict future observations.

Many different equation structures are possible under the broad framework of SSMs. More complex equation structures may better describe the underlying processes but can become more difficult or even intractable to fit. We use a simple structure for the observation and transition equations: linear dynamics with b noise. This formulation is a common approximation and has convenient mathematical properties. Concretely, let *y_t_* be the response to a particular EMA question on day *t* and *x_t_* be the hidden state on that day. This gives the following equations:

y_t_ = ax_t_ + c + v_t_ (observation equation) **(1)**

x_t__+1_ = bx_t_ + d + w_t_ (transition equation) **(2)**

where *v_t_* and *w_t_* represent independent, zero-mean Gaussian noise at each time step. Concretely, this structure states that on day *t*, the response to an EMA question (or the chance of lapse) is a linear function of the participant’s current hidden mental state plus random noise. The participant’s mental state the following day, *t+*1, is a separate linear function of their current mental state plus random noise. More generally, the observable quantities (EMA responses and lapse behavior) are expressions of the hidden state (mental constructs relevant to lapse), which evolve noisily over time. Model fitting identifies the joint “story” (ie, the observation and transition equations) that best explains the individual’s observed behavior over time. Predictions are straightforward to obtain by simulation, for instance, by “playing forward” the transition equation over the desired horizon and passing the state quantities through the observation equation. For additional details, see Section S3.5 in Multimedia Appendix 1.

Note that this approach is inherently personalized, as the parameters *a*, *b*, *c*, *d*, and noise variance are estimated separately for each participant rather than for the overall population. All participants have the same linear-Gaussian model structure, but the parameters are estimated individually to create a personalized model that best explains their observed behavior. Importantly, since these fitted equations directly use the observed EMA responses and lapse values, this approach does not require the manual creation of features. We detail the vector forms of these equations used to model all 9 EMA questions and lapse in Section S3 in Multimedia Appendix 1.

Fitting SSMs of this form is computationally inexpensive, so it is feasible to improve parameter estimates over time by refitting the model for each participant with each new day of available data. A common approach for fitting such models is maximum likelihood estimation (MLE) [52]. MLE identifies the parameters that best explain the participant’s behavior, but this approach is problematic for model fitting with rare outcomes. If a participant has never been observed to lapse, the best fitting parameters will be those that suggest they will not lapse in the future either. To address this, our modeling uses a Bayesian fitting approach called maximum *a posteriori* estimation [53], which incorporates prior distributions for the model parameters. The resultant fit is one that best explains both the participant’s observed data and the parameter priors, leading to models that better anticipate lapse risk even when the participant has never before lapsed. When limited data for the participant are available, the fit relies heavily on the prior distributions for the model parameters. The influence of these prior distributions diminishes as more data become available and the model fit primarily reflects the participant’s data. The prior distributions used in this paper were found by fitting models using MLE on the full trajectories of other participants within this studied cohort, gathering the set of estimates for each parameter, and fitting well-behaved distributions to those parameter sets. For a detailed explanation, see Section S3.6 in Multimedia Appendix 1.

Note that this approach combines idiographic and nomothetic principles. For instance, fitting a model to each participant is fundamentally idiographic, but this process incorporates nomothetic principles as well. First, this approach assumes that the behavior of all participants can be well-approximated by a common linear-Gaussian model structure built from observations of the same set of EMA questions. Further, we incorporate information from other participants through the parameter prior distributions. These priors are built from the results of MLE model fits for other participants. This means that we begin by assuming that participants behave similarly (ie, have similar parameter values) but personalize these model fits as data for each participant become available. We provide thorough descriptions of SSM fitting, implementation, prediction, creation of priors, and limitations in Section S3 in Multimedia Appendix 1.

### Benchmark ML Methods

We compared SSM performance to that of LR and gradient boosted classifiers. LR was implemented using Scikit-learn [54], and gradient boosting was implemented with extreme gradient boosting (XGB) [55]. Both LR and XGB used a common feature set given in Textbox 3 and the lapse labels described previously.

**Features used in the logistic regression and extreme gradient boosting models.**

**Features**

- Day of the week: Expressed as one-hot encoding for each day of the week. One of the 7 Booleans was dropped for logistic regression to avoid collinearity issues.
- Most recent ecological momentary assessment (EMA) responses: The ordinal responses to the most recent available morning EMA, scaled to [0,1]. As noted previously, the first morning EMA question dealt with reporting previous lapses, so these features correspond to the 9 ordinal responses provided from EMA questions 2-10.
- Short-run mean of EMA responses: The average of the participant’s responses in the 3 most recent available morning EMAs (9 questions), scaled to [0,1]. Fewer EMAs were used to calculate these features when 3 EMAs were not available.
- Long-run mean of EMA responses: The average of the participant’s responses to all available morning EMAs in their study period up to the current day (9 questions), scaled to [0,1]. For the data availability analysis, this average was calculated using smaller intervals (eg, the most recent 15 days of data).
- Recent lapses: Three Booleans indicating whether the individual had lapsed on any day in the last 1, 3, and 5 days.

Given the difficulty of fitting classification models for rare outcomes, these models were fit at the population level. On one hand, this is a fundamentally nomothetic approach as it creates 1 model mapping features to risk for the entire population. However, this approach allows for some measure of personalization through the model’s features. For instance, by including calculations of short- and long-run mean values for each EMA question as features, the model can capture risk associated with deviations from an individual’s baseline response level. For additional technical details, see Section S4 in Multimedia Appendix 1.

### Fitting Procedures

The SSMs in this study are time-series models trained for each participant, meaning they have inherently different fitting procedures than the population-trained benchmark ML classifiers. This section describes the idea of data availability used to assess the impact of model personalization and explains the fitting procedures used for each method.

#### Data Availability

A model’s ability to personalize to an individual becomes relevant as more of that individual’s data become available. The SSMs are explicitly personalized, with a separate model fit to each participant. While the benchmark ML classifiers are fit at the population level, they allow for limited personalization through feature construction. We evaluated the impact of model personalization on performance by varying what we call “data availability,” which is the number of days of EMA data available to a model from the test participant. For the benchmark ML models, this meant varying the number of days of EMA data available for feature construction. For the SSMs, which were trained separately for each individual, this meant varying the number of days of data used to train the model. To make this notion more concrete, we provide examples in the training procedures below for each model type.

#### LR and XGB

LR and XGB were trained and evaluated using 15 repeats of 5-fold nested cross-validation. In each repeat, the dataset was divided into 5 participant-stratified folds. We relied on scikit-learn’s StratifiedGroupKFold function [54]. This function forms folds such that the overall proportion of positive labels is similar across folds, and all data for each participant are contained in exactly 1 fold. Each fold was used once for testing, with the remaining 4 folds used for hyperparameter tuning and model training. We tuned model hyperparameters by grid search, using 5-fold participant-stratified cross-validation nested within the training folds. Additional details regarding hyperparameter tuning can be found in Section S4.2 in Multimedia Appendix 1. The model was refit on the training folds using the best performing hyperparameters and tested on the held-out fold. To assess how the performance of LR and XGB changed with respect to data availability, we created separate featurizations for the test fold using 15, 30, 45, 60, and 75 days of data availability. For example, the 15-day test set included only study days from day 15 onward, and its features were constructed with only the most recent 15 days of data (ie, the long-run average EMA features for day 70 included only data from study days 56-70). In contrast, the 45-day featurization for day 70 used data from days 26-70 to calculate long-run average values. These alternative featurizations were used only at test time and not for model training. Models were trained using the full study periods of all participants in the training folds.

#### Approach Involving SSMs

**Figure 2:**
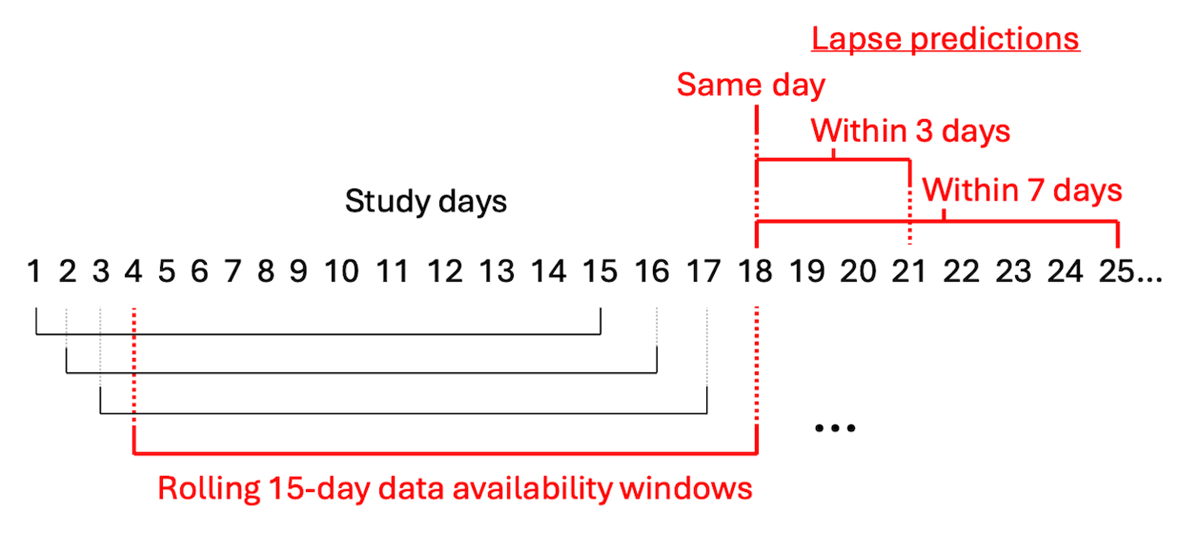
Illustration of the data availability fitting procedure used for state space models in this study. Data availability refers to the amount of the participant’s data that is available to use when training the model. As an example, this graphic considers 15 days of data availability, leading to models trained with data from days 1-15, 2-16, 3-17, and so on. The red bracketed case describes the scenario where a model is trained on ecological momentary assessment data from days 4-18 and lapse data from days 4-17. The trained model is used to make a same-day lapse prediction for day 18 and 2 window-style predictions for day 18 (ie, a lapse between days 18-21 and 18-25). This fitting procedure is repeated for each participant for different data availabilities (15, 30, 45, 60, and 75 days).

The SSMs were fitted using the same 15 repeated 5-fold cross-validation splits as the LR and XGB models. However, SSMs differ from population-trained models in that they are idiographic, with a different model trained for each participant. For each iteration within a cross-validation repeat, fitting proceeded as follows. Data from the participants in the 4 training folds were used to create prior distributions for the SSM parameters. We then fit an idiographic model to each participant in the test fold using their EMA and lapse data. To assess how the SSMs performed with respect to data availability and provide a direct comparison to LR or XGB predictions, we used rolling training periods of fixed length (15, 30, 45, 60, and 75 days). Figure 2 provides a diagram illustrating this fitting procedure. This fitting process created a set of predictions from the SSMs that was directly comparable to the LR or XGB predictions. While the fitting procedures differed between models, they had access to the same data and made predictions for the same set of tasks each day. For example, consider the 15-day case. Each model type produced predictions for each participant’s study day from day 15 onward. For the SSMs, predictions were made with models trained on the most recent 15 days of EMA and lapse data. For LR and XGB, the models were trained on participants from the 4 training folds and tested using the 15-day featurizations of EMA and lapse data.

### Statistical Analysis of Model Comparisons

Although fitting procedures differed between the models, they generated predictions that could be used to directly compare model performance. We gathered all predictions made for each test fold to create a receiver operating characteristic curve and summarized performance using the area under the receiver operating characteristic curve (AUROC) [56]. We focused our analysis on AUROC because we plan to directly use the continuous probability scores output by the models to inform treatment strategies and not to make a classification decision involving a particular decision threshold. However, other performance metrics, for instance, the area under the precision-recall curve (AUPRC), provide additional value in interpreting model performance [57]. For all assessments presented in terms of AUROC, we provide comparable assessments by AUPRC in Section S6.3 in Multimedia Appendix 1.

We compared the models using Bayesian hierarchical modeling [58,59] to quantify uncertainty in different aspects of the experiments, namely separating uncertainty in mean model AUROC performance from uncertainty in the cross-validation process. In this approach, the AUROC performance of a model on cross-validation repeat *i* and test fold *j* was modeled using a generalized linear model with a logit link function as follows:



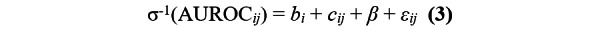



where *b_i_* is a cross-validation repeat intercept, *c_ij_* is a fold-within-repeat intercept, *β* is the model type’s mean AUROC performance, and *ε_ij_* is zero-mean Gaussian noise. This structure implies that each method has logit-transformed Gaussian mean AUROC performance, but that the mean is shifted based on the specific repeat and fold of the cross-validation process. The repeat intercept implies that, across all models, the test folds within a given repeat are expected to show similar AUROC performance since the training folds share large amounts of training data. The fold-within-repeat intercept reflects that common AUROC trends are expected for each test fold (ie, easier or harder across all methods). Analysis was performed using the TidyPosterior package [60]. Additional details are provided in Section S5 in Multimedia Appendix 1.

## Results

### Study Cohort Demographics

The collected demographic information for the study cohort is provided in Table 1.

**Table 1:** Demographic information of the alcohol use disorder study participants considered in our modeling (N=148).

Characteristic		Value
Age (years), mean (SD)		40.8 (11.9)
**Sex, n (%)**		
	Male	76 (51.4)
	Female	72 (48.6)
**Race, n (%)**		
	American Indian/Alaska Native	3 (2.0)
	Asian	2 (1.4)
	Black/African American	7 (4.7)
	White/Caucasian	129 (87.2)
	Other/Multiracial	7 (4.7)
**Hispanic, Latino, or Spanish origin, n (%)**		
	Yes	4 (2.7)
	No	144 (97.3)
Personal income (US$), mean (SD)		34,249 (31,517)
Number of quit attempts, mean (SD)		8.6 (31.0)
DSM-5^a^ alcohol use disorder symptom count, mean (SD)		8.8 (1.8)

^a^DSM-5: Diagnostic and Statistical Manual of Mental Disorders, Fifth Edition.

### EMA Adherence and Lapses

**Figure 3:**
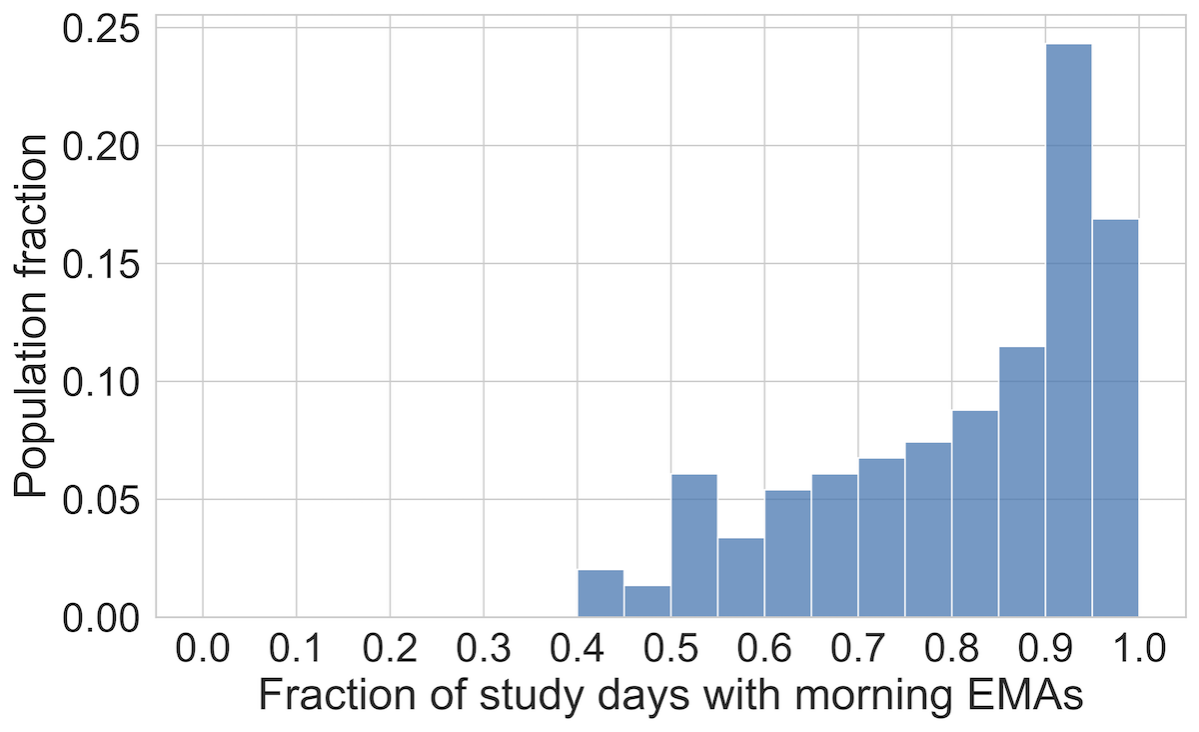
Histogram describing the distribution of ecological momentary assessment (EMA) adherence for the complete study cohort. We characterize adherence as the proportion of study days that each participant responded to the EMA after being prompted each morning. The median adherence was approximately 0.86.

**Figure 4:**
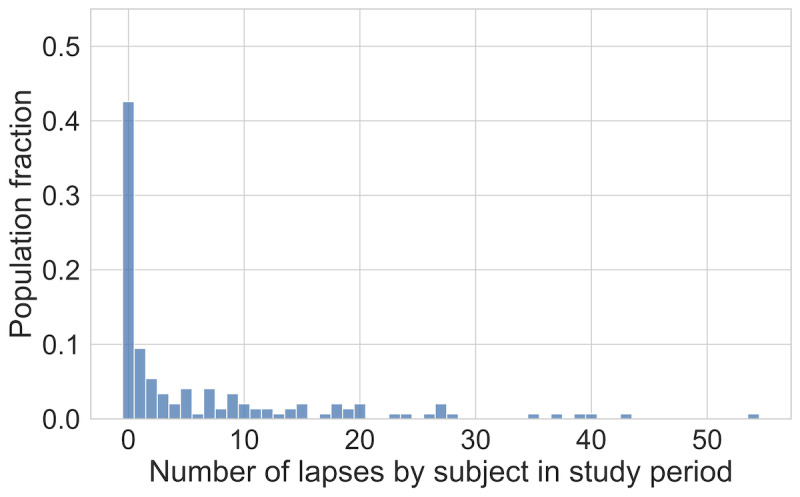
Histogram describing the distribution of total lapses during the study for the study cohort. Specifically, this histogram summarizes the fraction of the study cohort that reported each number of total lapses for the complete study period. Note that nearly 45% of participants reported no lapses during the study. The median lapse count for a participant was 1.

We measured EMA adherence as the proportion of study days that each participant completed a morning EMA. The distribution of adherence across the study population is shown in Figure 3. Lapses were relatively rare, with approximately 7.5% of study days containing lapses across the study population. The distribution of total lapses by participants in the study period is shown in Figure 4.

### Lapse Prediction Performance

**Figure 5:**
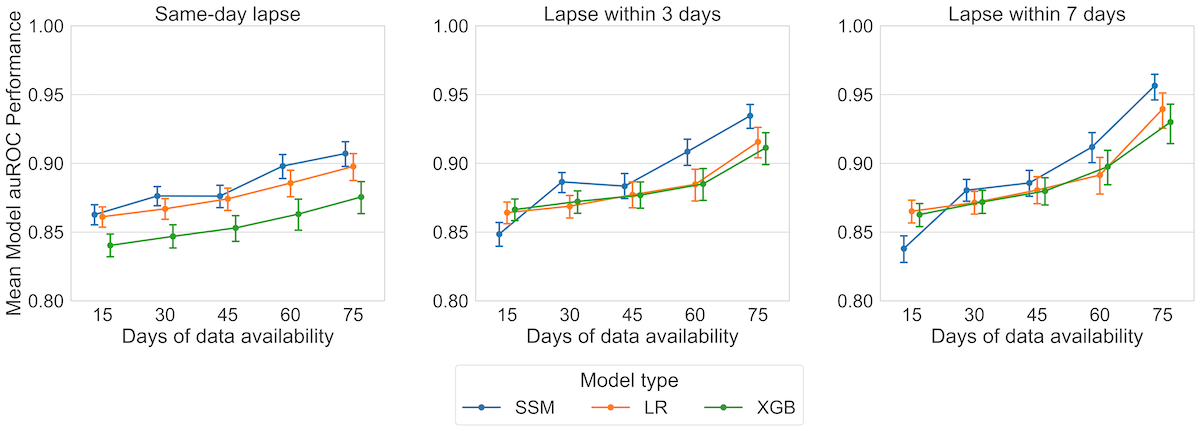
Plots of posterior mean area under the receiver operating characteristic curve (AUROC) performance for 3 prediction tasks and 3 model types (state space model [SSM], logistic regression [LR], and extreme gradient boosting [XGB]). Medians are marked with dots, and 95% credible intervals (CrI) are provided as error bars. Each panel describes performance on a different prediction task (ie, same-day lapse, lapse within 3 days, and lapse within 7 days). The values reported for the 3 models at each x-value (number of days of data availability) are offset slightly by method for easier viewing of the median and CrIs. Note that there is substantial correlation between the performance of the different models in the posterior samples, making visual ranking comparisons using the CrIs incomplete. Model ranking is instead assessed by a separate summary calculation of posterior samples that accounts for this correlation.

Figure 5 shows the posterior mean AUROC performance obtained from Bayesian hierarchical modeling across prediction tasks and data availability.

Plots of the raw AUROC values from repeated cross-validation are provided in Section S6.1 in Multimedia Appendix 1. Table 2 provides a particular summary of the posterior distribution, specifically the posterior probability that SSMs had the best mean AUROC across all methods. These probabilities were calculated by drawing posterior samples of model performance from the Bayesian hierarchical model and identifying the fraction of samples with mean AUROC performance for the SSMs greater than that for LR and XGB. Note that additional posterior summaries discussed in the OSF preregistration are available in Section S5.3 in Multimedia Appendix 1.

Section S6.3 in Multimedia Appendix 1 contains plots and tables for AUPRC, which are analogous to those presented here for AUROC.

**Table 2:** A summary of state space model performance (posterior probability that the state space models had the highest mean area under the receiver operating characteristic curve across all methods) relative to the benchmark methods for each prediction task and amount of data availability.

Prediction task	Data availability (days)				
	15	30	45	60	75
Same-day lapse	0.732	0.999	0.760	0.999	0.995
Lapse within 3 days	<0.001	0.999	0.985	0.999	0.999
Lapse within 7 days	<0.001	0.997	0.913	0.999	0.999

## Discussion

### Principal Findings

We noted excellent performance across the tested methods, and our findings echo the sentiment of previously cited works [6,35-38]. The use of predictive modeling has the potential to meaningfully improve clinical treatment for AUD through accurate and automated risk assessment. Specifically, the experiments provide strong posterior evidence that SSMs can outperform existing ML approaches for AUD lapse prediction.

Figure 5 provides a coarse visual summary of mean AUROC performance, showing that the SSMs generally displayed higher mean performance than the benchmark methods. More formally, we rank-ordered the methods for each level of data availability using the posterior samples from our Bayesian hierarchical modeling. Table 2 provides the posterior probabilities that the SSMs had the best mean AUROC among all 3 assessed model types. We found strong evidence that, given 30 or more days of data availability, the SSMs outperformed the LR and XGB models. This held across all 3 prediction types, with the difference between the SSMs and LR or XGB models typically growing as data availability increased. There was significant posterior correlation between the mean AUROC performance of the different models. This explains why we obtained strong posterior evidence for the SSMs outperforming the other methods, even when there was overlap in the credible intervals for mean model AUROCs (Figure 5). A deeper discussion of this posterior correlation is provided in Section S6.2 in Multimedia Appendix 1.

More broadly, these results suggest a threshold of data availability occurring between 15 and 30 days of data availability, which divides 2 relevant performance regimes. Above this threshold, we noted uniformly better performance across all tasks using the SSMs as compared to the benchmarks. Below this threshold, the performance results were mixed, with posterior estimates suggesting the SSMs had better same-day prediction performance but worse performance than the benchmark classifiers on longer prediction window tasks. We believe that this performance trend reflects the fact that SSMs are idiographic (training a unique model for each participant).

In the regime of low data availability (ie, only 15 days of data for a given participant), SSMs have very little patient-specific information to personalize a model beyond the initial parameter priors. SSMs estimate a model of each participant’s time-series behavior, and this single model generates predictions for all tasks by simulating trajectories of the behavior over 1, 3, or 7 days. This means that errors in the participant’s estimated model can be magnified when making predictions for longer time windows, likely explaining the poorer performance of SSMs on the latter prediction tasks.

As data availability increases, however, idiographic SSMs have more flexibility than population-trained ML benchmarks to capture the nuances of each participant’s behavior. Since a unique model is trained for each participant, it can learn personalized patterns of behavior without impacting predictive performance for the rest of the population. Once a critical amount of participant data is available, the benefits of personalization dominate and SSMs outperform benchmark approaches across all tasks.

These performance differences highlight the importance of idiographic modeling for AUD lapse risk. If there were no meaningful heterogeneity in the presentation of AUD symptoms and lapse behavior, we would not expect to see a gap between idiographic and population-trained models. Further, AUD is a chronic, relapsing condition calling for continuing care for months or years. While population-trained models may have superior performance in the initial weeks of observation (depending on the prediction task), the vast majority of treatment would occur in a regime of data availability favoring idiographic SSMs.

### Extensions to Intervention Frameworks

Beyond improved predictive performance, the use of SSMs enables exciting new approaches to treatment selection. SSMs build an approximate dynamic system model for how an individual’s state of mind evolves over time. Using this, we can draw on powerful mathematical tools like control theory [61-65] to guide treatment decision-making. Control problems have 3 core components: a system model, a set of possible actions to take, and a defined objective. For example, in the case of automobile cruise control, the control task is to choose the amount of acceleration or braking to comfortably maintain a particular speed or distance from a neighboring vehicle. Critical to calculating the optimal action is the dynamic system model, which describes how the vehicle will respond to a given amount of acceleration or braking [13].

With an approximate system model for a patient’s state of mind, we can apply a similar approach to treatment decisions. Using a digital therapeutics platform, a patient submits an EMA each morning. In response, the platform could automatically provide a risk estimate and a treatment recommendation to best support the patient’s recovery (eg, performing a mindfulness or treatment-related exercise, recalling a particular strategy to control craving, meeting a supportive friend for lunch, providing a reminder to avoid high-risk locations, etc). This type of automated support, which considers the personalized factors driving each patient’s risk, is a promising way to expand and improve care.

In principle, one could construct this type of treatment-recommendation platform using the benchmark classifier models, but SSMs offer a few specific advantages. First, as time-series models, SSMs allow for treatment recommendations that take the individual’s behavioral dynamics into account. In control theory, this notion is called *planning*. Choosing the optimal action considers both the state of the system today and how the system might evolve in the future (including its response to today’s chosen action). Interventions that myopically address factors causing elevated lapse risk *today* might be suboptimal choices to support the patient’s long-term recovery. As classifier models do not model the time series dynamics of a patient, they do not support this type of principled treatment selection.

Second, since SSMs are trained separately for each patient, they can capture the effectiveness of a treatment for an individual patient rather than for the overall population. To make this concrete, we recall the SSM transition equation. This equation describes how an individual’s hidden mental state (*x*) evolves over time, for instance, from day *t* to day *t+*1. Treatments would be included as additive terms in the transition equation:



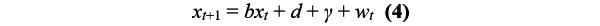



A *γ* term is added to the right-hand side and signifies the treatment effect of the chosen intervention. This treatment effect may differ between individuals and is thus an important consideration for how to optimally recommend treatments. Just as the parameters related to the individual’s behavioral dynamics, such as *b* and *d* here, are better estimated over time, we can similarly learn the personalized value of each treatment (*γ*) as additional patient data are collected. Note that this approach extends to cases where multiple possible interventions are considered. This would be expressed as a summation of distinct treatment effect terms (*γ_i_*), each multiplied by a binary decision variable reflecting the choice to recommend that treatment, such that only the selected treatments impact the patient’s system dynamics.

Further, a critical consideration in implementing effective digital therapeutic interventions is patient engagement [66]. The SSMs presented here can be augmented to include a state representing the participant’s capacity for engagement. This would allow the model to consider how engagement evolves over time and to incorporate this information in the treatment selection problem. For instance, a higher burden treatment exercise might only be effective when the patient has the mental capacity to engage fully with it, compared to when they are stressed or overwhelmed. By including engagement dynamics in the patient model, the digital therapeutic platform could better recommend treatments to support patient recovery.

### Clinical Interpretability of Modeling Approaches

Broadly, we believe that SSMs have the potential to provide improved clinical interpretability over the tested benchmark approaches, specifically through their personalization and the modeling of patient dynamics. To make these notions more concrete, we consider interpretability approaches for each model type.

LR models offer significant interpretability, as a clinician can directly evaluate fitted model coefficients to understand how input features impact risk assessment, and further examining the patient’s specific covariates surrounding lapses can provide insights into what factors are driving lapse risks for a particular patient. Importantly, however, these interpretations are limited when models are fit at the population level, as the model coefficients are not necessarily representative of an individual patient.

Boosted tree-based models like XGB typically offer improved predictive performance but are less directly interpretable than an LR model. To understand which features are driving a patient’s risk, a clinician might employ feature explainability tools like Shapley values [67]. These provide a way to assess the contribution of a particular feature (or groups of features) to predictions. Similar to LR, these values are somewhat limited when models are fit at the population level rather than to individuals.

SSMs are similar to LR models in that a clinician can directly examine coefficient values within the observation equations to see how particular mental constructs affect lapse risk. Analyzing specific latent state values can further inform which constructs are driving a patient’s lapse risk. Importantly, since SSMs are fit to each individual, these coefficients reflect the behavior of a specific patient rather than the overall population. Additionally, SSMs capture the dynamics of these mental constructs through the transition equation. For instance, a patient’s dynamics might indicate that one mental construct tends to decay from elevated levels toward baseline levels very slowly compared to another more transient construct (these quantities are directly visible as fitted parameters within the transition equations). Even if the current values of these example constructs contribute similarly to immediate risk, a clinician might choose to address the slower-decaying construct to better mitigate both immediate and longer-term lapse risk. This type of insight is not possible without a model that captures an individual’s time-varying dynamics.

### Limitations and Opportunities

The SSMs and study data presented in this manuscript have several limitations and considerations relevant for future work. We provide a discussion of the broader limitations here and point technically interested readers to additional modeling discussions in Section S3 in Multimedia Appendix 1.

#### Limited Demographic Diversity in the Study Population

This study is limited by its consideration of a narrow geographical area with correspondingly limited demographic diversity. This is typically a concern because trained models may show poor performance in populations that are underrepresented in the training sample [68,69]. We believe that such fairness concerns may be partially alleviated by the type of idiographic modeling framework presented here. Rather than applying a single, population-trained model to everyone, we advocate fitting personalized models to each patient that capture their unique patterns of behavior. However, whether this approach truly alleviates such concerns will require validation in separate studies. Evaluating the potential algorithmic fairness benefits of personalized models will be the focus of future work.

#### Limited Interpretability of SSMs due to Latent State Definition

The SSMs presented here have specific interpretability limitations, but these are not necessarily reflective of SSM approaches in general. In principle, SSMs can be constructed such that each latent state represents a specific mental construct. In this case, it is helpful to carefully structure EMA questions to provide targeted measurements of each relevant construct. Our modeling was performed after the parent study was designed and completed, and thus, we could not influence the existing EMA format. As such, we chose to use 2 latent states with no specific interpretations. This demonstrates that it is possible to approximate behavior well even with a low-dimensional linear model but indicates that this work cannot highlight the additional explanatory benefits of SSMs relative to classifier methods. We hope that future work will explore interpretable latent structures to bring SSMs closer to the existing literature of explanation-focused psychological modeling. Doing so would allow clinicians to examine fitted models to improve personalized treatment planning with patients [70].

#### Proposed SSMs Assume Time Invariance for Patient Models

The SSMs we presented assume that participants’ dynamic models are time invariant. Over the 3-month study period, this may be a plausible assumption but is a clear limitation for longer time horizons. If individuals are trending toward recovery, this change should be represented through time-varying model parameters. For example, while craving might strongly influence lapse early in a participant’s recovery, these dynamics may change over time as the participant learns to better address cravings, which can diminish their influence. SSMs can be structured to model these types of time-varying parameters [71], but this structure would require modifications to the model estimation strategies presented in this manuscript.

#### Performance Results Should be Considered in the Context of the Study Cohort

The predictive performance values achieved by our SSMs and the benchmark methods were linked to the size and composition of the study cohort as well as our chosen cross-validation structure. In the case of SSMs, the study cohort size and cross-validation fold count affected the number of other participants included in the fitting of prior distributions for individuals in the test fold. As our prior distributions were entirely data-driven, this effective cohort (ie, the count and composition of the individuals in the training folds) determined the strength of the priors constructed for the test fold. For the benchmark methods, the cohort size and cross-validation fold count determined the size of the model training set. In future work, we plan to examine larger participant cohorts to rigorously characterize the impact of these factors on model performance.

#### Actively Collected Patient Data Have Important Limitations and Potential Biases

EMA data collection naturally imposes a burden on participants, although EMA data collection once per day, as used in this study, appears to be well-tolerated by patients for monitoring over months or years [43,47]. We thus believe that the performance reported in this study sets a plausible benchmark for the real-world use of such methods. However, we note that we excluded 3 of 151 participants from the original study based on poor EMA compliance (responding to fewer than 25% of prompted EMAs), as EMA noncompliance can introduce problems for all tested methods. Without reasonably up-to-date patient data, the prediction problem is not well-posed. While this decision represents a relatively small exclusion, it does mean that our results are more representative of compliant individuals. The use of passively collected data, such as GPS or sleep quality data, will be the focus of future work, as this may provide a means of gathering sufficient patient data for prediction, even with limited EMA compliance.

Further, while EMA represents an important step forward in patient data collection, it is still important to consider its structural limitations. For instance, as noted above, the primary use of EMA data introduces the possibility of participant selection bias [72], participant responses may be influenced by social desirability factors [73], and data quality may suffer from inattentive or careless responding [74]. Despite these potential limitations, we are encouraged to find that all tested models still performed well in our testing. We believe that the integration of passively collected data can help address the limitations of EMA data and make predictive systems more robust.

None of the modeling approaches (SSM, LR, or XGB) in this study incorporated passively sensed information, such as GPS, but all could, in principle, be modified to do so. For the LR or XGB models, integrating passively collected data would require the creation of new model features. For SSMs, integrating passive data would involve adding elements to the observation equations that relate passively observed quantities, such as the fraction of time spent in low-risk locations versus high-risk locations, to new functions of the latent states. We plan to incorporate passively sensed data in future work to decrease the sensing burden on participants, potentially improve prediction quality, and mitigate sources of bias.

#### Extensions to Other Mental Health Conditions

This work presents a case study for the use of SSMs in the context of AUD lapse prediction. The promising performance of SSMs in this domain suggests that they merit examination for risk assessment tasks in other mental health conditions (eg, predicting panic attacks or suicidal ideation). Of course, whether SSMs similarly outperform the benchmark ML approaches for other conditions will require validation in separate studies. It may be that, for instance, the benefits of personalization are more pronounced in some conditions versus others. For conditions with minimal patient heterogeneity, we would expect all approaches (SSM, LR, and XGB) to have similar predictive performance. However, even in these cases, we believe the structural benefits of SSMs (eg, easier integration into intervention frameworks for treatment selection) may make them a compelling choice for clinicians.

From an implementation standpoint, both SSMs and the benchmark ML approaches (LR and XGB) would require similar modifications to adapt them to new risk assessment tasks. SSMs model observable quantities (eg, EMA responses or clinically relevant events like lapse for AUD) as functions of dynamic processes of unobserved quantities (eg, a patient’s evolving state of mind). Applying this framework to new conditions would involve determining which observable quantities are relevant to the condition under study. For instance, this might involve a different set of EMA questions (which could be smaller or larger than the 9-question EMA in this study) and a different primary prediction target (eg, panic attacks rather than AUD lapses). This is similar to the changes needed to apply LR or XGB approaches to a new condition. These models would also require potentially different EMA data sources, redefinition of appropriate features, and redefinition of the prediction target.

It is possible that different mental health conditions would require further structural modifications to the tested models. For the benchmark ML methods, this could entail different strategies in feature construction or the exploration of different model hyperparameters. Structural modifications to SSMs could involve changes, such as a larger or smaller number of latent states, a different granularity in time discretization, or modifications to the structure of the transition equations. These changes may drive modifications to the fitting approaches proposed in this manuscript but would not alter the types of predictions that can be made or the possible time series and personalization benefits of SSMs over the benchmark approaches. To begin exploring these questions more deeply, we plan to validate this approach in other forms of SUD prediction, such as lapses in opioid use.

### Conclusion

This work presents a framework for the use of SSMs to predict lapses in individuals with AUD. Using a 3-month study of 148 individuals in early recovery from AUD, we demonstrated that SSMs can offer better lapse prediction performance compared to benchmark ML methods used in the literature. We have discussed the additional benefits of state SSMs as time-series methods. In particular, time-series approaches bring prediction-focused work closer to the methodologies used in explanation-focused psychological modeling and enable exciting new continuing care approaches using digital therapeutics. While our work deals specifically with AUD, we believe that the same modeling principles are suitable for evaluation in a variety of pressing mental health conditions. New approaches for automated risk monitoring and treatment suggestion can both improve care and help extend care to undertreated populations.

## Appendix

Multimedia Appendix 1 Additional technical information.

Multimedia Appendix 2 Transparency report.

Multimedia Appendix 3 Checklist for machine learning predictive modeling in biomedical applications.

## Abbreviations

AUD: alcohol use disorder
AUPRC: area under the precision-recall curve
AUROC: area under the receiver operating characteristic curve
EMA: ecological momentary assessment
LR: logistic regression
ML: machine learning
MLE: maximum likelihood estimation
OSF: Open Science Framework
ROC: receiver operating characteristic
SSM: state space model
SUD: substance use disorder
XGB: extreme gradient boosting

## Data Availability

We have provided all data necessary to reproduce the reported lapse prediction performance results at our publicly available, persistent project repository on Open Science Framework (see [41]). This repository also contains the code used for model fitting and the Bayesian analysis of predictive performance. Participant-level data are not included in this repository in order to safeguard protected health information. To discuss access to these data, please contact JC.
