# Supplementary material for: Idiographic Lapse Prediction With State Space Modeling: Algorithm Development and Validation Study
Source: JMIR Form Res. 2025 Jun 3;9:e73265. doi: 10.2196/73265 (PMC12174888; doi:10.2196/73265)
Supplement: Multimedia Appendix 1 [file formative_v9i1e73265_app1.pdf]

Contents

S1 Overview 2

S2 Study participant information 2

S3 State space models 2
- S3.1 State space model preliminaries . . . 2
- S3.2 Fitting overview . . . 3
- S3.3 Maximum likelihood estimation . . . 4
  - S3.3.1 A update . . . 6
  - S3.3.2 B update . . . 6
  - S3.3.3 c update . . . 7
  - S3.3.4 d update . . . 7
  - S3.3.5 R update . . . 7
- S3.4 Maximum a posteriori estimation . . . 8
  - S3.4.1 A update . . . 9
  - S3.4.2 B update . . . 9
  - S3.4.3 c update . . . 10
  - S3.4.4 d update . . . 11
  - S3.4.5 R update . . . 11
- S3.5 Generating lapse predictions . . . 12
- S3.6 State space model prior distributions . . . 13
- S3.7 Implementation notes . . . 14
- S3.8 Modeling limitations and considerations . . . 15

S4 Machine learning models 15
- S4.1 Feature construction . . . 15
- S4.2 Hyperparameter tuning . . . 16
- S4.3 Fitting scripts . . . 16

S5 Statistical analysis 16
- S5.1 Overview . . . 17
- S5.2 Hierarchical modeling priors . . . 17
- S5.3 Additional posterior reporting . . . 17

S6 Additional plots 19
- S6.1 Raw auROC values . . . 19
- S6.2 Posterior mean auROC correlation . . . 19
- S6.3 Area under the precision-recall curve . . . 21

References 22

S1 Overview

This appendix provides additional details for the study cohort, state space modeling, machine learning modeling, statistical analysis, and supplementary plots.

S2 Study participant information

Demographic information, EMA adherence, and lapse prevalence for the study cohort are described in the main manuscript. Patients were compensated for their participation in the study, based on adherence to a number of study measures, some of which were not included as part of the modeling in this manuscript (e.g., sleep assessment and recording audio journal logs). The complete compensation information is available in the file `study_documents/Consent_IRB_2018_1005.pdf` on OSF, specifically under the section titled "WILL I BE COMPENSATED FOR MY PARTICIPATION?" beginning on page 4. For any further details regarding the study cohort, we direct readers to the supplementary materials provided by Wyant et al. [21].

S3 State space models

State space models represent a large class of modeling approaches for time series data [6]. This manuscript considers a very well studied subclass: linear-Gaussian models [18]. This means that the observations (EMA responses and binary lapse label) on a particular day are modeled as linear functions of the hidden state on that day plus zero-mean Gaussian noise. Similarly, the hidden state for the next day is modeled as a linear function of the current hidden state plus zero-mean Gaussian noise. We stress that this linear-Gaussian approach is an approximation for participants' mental dynamics and is not proposed as a causal model.

This section contains a mathematical description of the state space models used in this project, along with procedures for fitting model parameters by maximum likelihood estimation and maximum *a posteriori* estimation.

S3.1 State space model preliminaries

We begin by defining the following quantities:

- Ecological momentary assessments (EMA) contained 10 questions (i.e., Q1-Q10). EMA Q1 deals with reporting of lapses and is not included as part of this model. Responses to the remaining questions are discrete and range from either 0-12 (EMA Q2-Q5) or 1-11 (EMA Q6-Q10). For simplicity, we re-index such that Q2 corresponds to the first element of the response vector. Let $\tilde{\boldsymbol{y}}_t^k$ be the observed survey response vector of subject k at time step t where $\tilde{y}_{i,t}^k$ is the response to question $i = 1, \dots, 9$. Time steps $t \in \{1, \dots, T_k\}$ represent the day index in the study period (i.e., $T_k = 90$ corresponds to an individual who participated for the complete 3-month study period). The paraphrased Likert-style EMA questions are as follows:

  $\tilde{y}_1$: Since your last survey how intense was your greatest urge to drink alcohol?

  $\tilde{y}_2$: Since your last survey did you encounter any risky situations? Rate the most intense one.

  $\tilde{y}_3$: Since your last survey has a hassle or stressful situation occurred? Rate the most intense one.

  $\tilde{y}_4$: Since your last survey has a pleasant or positive event occurred? Rate the most intense one.

  $\tilde{y}_5$: How are you feeling right now (unhappy to happy)?

  $\tilde{y}_6$: How are you feeling right now (calm to aroused)?

  $\tilde{y}_7$: How likely are you to encounter risky situations within the next week?

  $\tilde{y}_8$: How likely are you to encounter stressful events within the next week?

$\tilde{y}_9$: How likely are you to drink alcohol within the next week?

- Let $e_t^k \in \{0, 1\}$ be the binary outcome of whether subject k had a lapse event on time step t. Note that we define a day to run from 4AM-4AM such that days are better aligned with continuous time stretches between sleep.
- Let $\boldsymbol{x}_t^k$ be the vector of unobserved states for subject k at time step t where $x_{j,t}^k$ is the jth unobserved state component ($j = 1, \ldots, m$).

Our model assumes linear dynamics with respect to the latent states $\boldsymbol{x}$ and Gaussian noise for both the EMA responses and the binary lapse outcome.[1] We define $\boldsymbol{y}_t^k$ to be the complete observation ($n \times 1$) for subject k at time t, given by:

$$\boldsymbol{y}_t^k = \begin{bmatrix} y_{1,t}^k & y_{2,t}^k & y_{3,t}^k & y_{4,t}^k & y_{5,t}^k & y_{6,t}^k & y_{7,t}^k & y_{8,t}^k & y_{9,t}^k & e_t^k \end{bmatrix}^T \tag{1}$$

where $y_{i,t}^k$ denotes the response to EMA question i, re-scaled to $[0, 1]$.

We can thus form the state space model for the kth individual (dropping the k superscripts for brevity):

$$\boldsymbol{y}_t = \mathbf{A}\boldsymbol{x}_t + \boldsymbol{c} + \boldsymbol{v}_t \qquad \boldsymbol{v}_t \sim \mathcal{N}(0, \mathbf{R}) \; \forall t \tag{2}$$

$$\boldsymbol{x}_t = \mathbf{B}\boldsymbol{x}_{t-1} + \boldsymbol{d} + \boldsymbol{w}_t \qquad \boldsymbol{w}_t \sim \mathcal{N}(0, \mathbf{Q}) \; \forall t \tag{3}$$

$$\boldsymbol{x}_1 \sim \mathcal{N}(\boldsymbol{\mu}_1, \boldsymbol{\Lambda}) \tag{4}$$

Where $\mathbf{A}$ is an $n \times m$ matrix, $\mathbf{B}$ is an $m \times m$ matrix (assumed to be diagonal), $\boldsymbol{c}$ is an $n \times 1$ vector, $\boldsymbol{d}$ is an $m \times 1$ vector. $\boldsymbol{v}_t$ and $\boldsymbol{w}_t$ are n and m dimensional Gaussian noise vectors, assumed to be time invariant. $\mathbf{R}$ is an $n \times n$ matrix (assumed to be diagonal), $\mathbf{Q} = \mathbf{I}_m$, $\boldsymbol{\mu}_1$ is the zero vector, and $\boldsymbol{\Lambda} = \mathbf{I}_m$. Note that in this iteration of the model we fix the variance associated with lapse to 0.25, but plan to explore fitting the lapse process variance in future iterations of the model. Here $n = 10$ (9 questions plus a lapse outcome) and $m = 2$. The complete SSM can be represented as a graphical model with the latent state each day producing EMA and lapse emissions, as given in Figure 1.

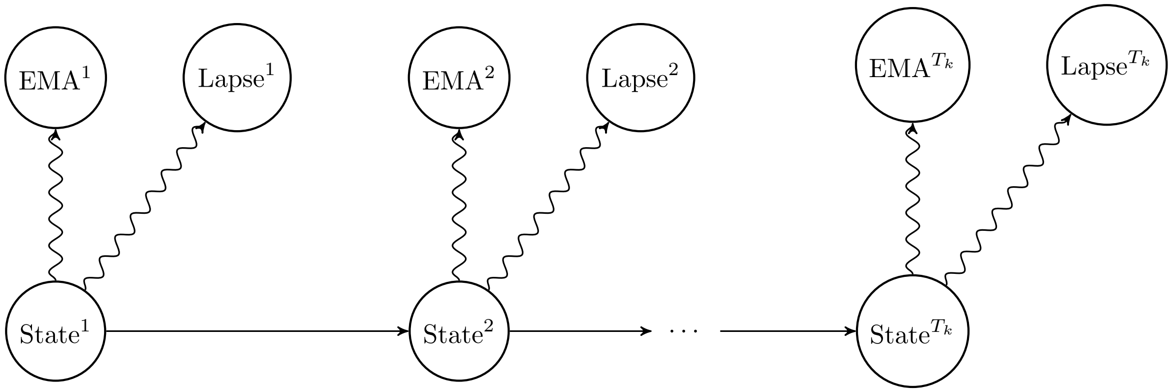

Figure 1: A graphical representation of the state space model. Latent states evolve in time according to the system's transition equation (Eqn. 3) and produce EMA and lapse observations according to the system's observation equation (Eqn. 2).

S3.2 Fitting overview

These state space models can be fit by either maximum likelihood estimation (MLE) or maximum *a posteriori* (MAP) estimation. In the case of MLE, only the participant's data is used to estimate their model parameters. MAP estimation is similar, but uses prior distributions over the model parameters as part of the estimation process. In this project, we use MLE to fit models to participants in the training dataset, which

[1]This is an approximation that requires clipping for prediction, as this model structure does not limit predictions to the interval (0,1). We discuss this and other limitations in Section S3.8.

are then used to create prior distributions for use in MAP estimation of the test individual's model parameters (see Section S3.6). As data availability approaches infinity the two estimation approaches converge to the same model, but incorporating priors for model parameters helps these models make better predictions when fewer data are available.

Both approaches rely on the Expectation-Maximization algorithm [5]. Broadly, this approach involves alternatively fixing one of the latent state trajectory and the model parameter set then optimizing over the other. This process results in an improved model fit each iteration and the process is repeated until improvements in the model fitting criterion fall below a user-defined threshold.

S3.3 Maximum likelihood estimation

This section describes the process of fitting model parameters using MLE. Portions of this section closely follow the technical documentation for the `MARSS` package [9]. Also note that we present the derivations for the case of no missing data for brevity, but that our implementation is extended to the missing data case as given in [9].

In the MLE fitting process, we wish to find the model parameters $\boldsymbol{\theta}$ that maximize the likelihood function for each individual, defined as:

$$\mathcal{L}(\boldsymbol{\theta}) = f(\boldsymbol{y}|\boldsymbol{\theta}) \tag{5}$$

where $\boldsymbol{y} = (\boldsymbol{y}_1, \ldots, \boldsymbol{y}_T)$ is the sequence of observation vectors. Similarly let $\boldsymbol{x} = (\boldsymbol{x}_1, \ldots, \boldsymbol{x}_T)$ be the sequence of latent state vectors. We expand the likelihood to include marginalization of the distribution of hidden states:

$$f(\boldsymbol{y}|\boldsymbol{\theta}) = \int_{\mathbf{X}} f(\boldsymbol{y}, \boldsymbol{x}|\boldsymbol{\theta}) d\mathbf{x} \tag{6}$$

The parameter set which maximizes the likelihood equivalently maximizes the log-likelihood, $\ell(\boldsymbol{\theta})$. Let $Q(\boldsymbol{x})$ be an arbitrary probability distribution for the sequence of latent states. We have:

$$\begin{aligned}
\ell(\boldsymbol{\theta}) = \log\left(\mathcal{L}(\boldsymbol{\theta})\right) &= \log\left(\int_{\boldsymbol{X}} Q(\boldsymbol{x}) \frac{f(\boldsymbol{y}, \boldsymbol{x}|\boldsymbol{\theta})}{Q(\boldsymbol{x})} d\boldsymbol{x}\right) \\
&= \log\left(\mathbb{E}_{x\sim Q}\left[\frac{f(\boldsymbol{y}, \boldsymbol{x}|\boldsymbol{\theta})}{Q(\boldsymbol{x})}\right]\right) \\
&\geq \mathbb{E}_{x\sim Q}\left[\log\left(\frac{f(\boldsymbol{y}, \boldsymbol{x}|\boldsymbol{\theta})}{Q(\boldsymbol{x})}\right)\right] \\
&= \mathbb{E}_{x\sim Q}\left[\log(f(\boldsymbol{y}, \boldsymbol{x}|\boldsymbol{\theta})\right] - \mathbb{E}_{x\sim Q}\left[\log(Q(\boldsymbol{x})\right]
\end{aligned} \tag{7}$$

where the inequality follows from application of Jensen's inequality. Note that for some fixed value of $\boldsymbol{\theta}$, we can select $Q(\boldsymbol{x})$ such that the inequality is tight. We do so by observing that Jensen's inequality must hold with equality when the associated function is constant [14], i.e. for some constants c, c', and c'':

$$\log\left(\frac{f(\boldsymbol{y}, \boldsymbol{x}|\boldsymbol{\theta})}{Q(\boldsymbol{x})}\right) = c \qquad \Rightarrow \qquad \frac{f(\boldsymbol{y}, \boldsymbol{x}|\boldsymbol{\theta})}{Q(\boldsymbol{x})} = c' \qquad \Rightarrow \qquad Q(\boldsymbol{x}) = c'' f(\boldsymbol{y}, \boldsymbol{x}|\boldsymbol{\theta}) \tag{8}$$

In order to ensure $Q(\boldsymbol{x}) = c'' f(\boldsymbol{y}, \boldsymbol{x}|\boldsymbol{\theta})$ is a proper distribution and integrates to 1, we normalize as follows:

$$\begin{aligned}
Q(\boldsymbol{x}) &= \frac{c'' f(\boldsymbol{y}, \boldsymbol{x}|\boldsymbol{\theta})}{\int_{\boldsymbol{x}} c'' f(\boldsymbol{y}, \boldsymbol{x}|\boldsymbol{\theta}) d\boldsymbol{x}} = \frac{f(\boldsymbol{y}, \boldsymbol{x}|\boldsymbol{\theta})}{f(\boldsymbol{y}|\boldsymbol{\theta})} \\
&= f(\boldsymbol{x}|\boldsymbol{y}, \boldsymbol{\theta})
\end{aligned} \tag{9}$$

implying that the inequality is tight when $Q(\boldsymbol{x})$ is chosen to be the posterior distribution for $\boldsymbol{x}$ given $\boldsymbol{y}$ and $\boldsymbol{\theta}$. The Expectation-Maximization algorithm maximizes $\ell(\boldsymbol{\theta})$ iteratively as follows:

Algorithm 1 Expectation-Maximization (MLE)

Input : Initial parameter iterate $\boldsymbol{\theta}_0$, observed data series $\boldsymbol{y}$

$k \leftarrow 0$

repeat

(E-Step) $Q_{k+1}(\boldsymbol{x}) \leftarrow f(\boldsymbol{x}|\boldsymbol{y}, \boldsymbol{\theta}_k)$

(M-Step) $\boldsymbol{\theta}_{k+1} \leftarrow \underset{\boldsymbol{\theta}}{\operatorname{argmax}}\, \mathbb{E}_{x\sim Q_{k+1}}\left[\log(f(\boldsymbol{y}, \boldsymbol{x}|\boldsymbol{\theta}_{k+1})\right] - \mathbb{E}_{x\sim Q_{k+1}}\left[\log(Q(\boldsymbol{x}))\right]$

$k \leftarrow k+1$

until converged

Note that $Q_{k+1}(\boldsymbol{x})$ is calculated using the current parameter iterate, but is not a function of $\boldsymbol{\theta}$. As a result, the argmax calculated during the M-Step can equivalently consider only the $\mathbb{E}_{x\sim Q_{k+1}}\left[\log(f(\boldsymbol{y}, \boldsymbol{x}|\boldsymbol{\theta}_{k+1})\right]$ term. Thus, the total log-likelihood, $\ell(\boldsymbol{\theta}) = \log(f(\boldsymbol{y}|\boldsymbol{\theta}))$, is maximized by maximizing the expected joint log likelihood, $\mathbb{E}\left[\log(f(\boldsymbol{y}, \boldsymbol{x}|\boldsymbol{\theta}))\right]$, in each iteration. The algorithm guarantees that the total log likelihood will not decrease from iteration to iteration [18], so convergence is defined once the increase in the total log-likelihood, measured between two iterations, falls below a given numerical tolerance.

For a system with linear dynamics and Gaussian noise, note that the E-Step can be calculated efficiently using the Kalman Filter and Rauch-Tung-Striebel (RTS) smoother [11, 17, 19]. We can expand the joint log-likelihood term using the definition of conditional probability:

$$\begin{aligned} f(\boldsymbol{y}, \boldsymbol{x}|\boldsymbol{\theta}) &= \frac{f(\boldsymbol{y}, \boldsymbol{x}, \boldsymbol{\theta})}{f(\boldsymbol{\theta})} = \frac{f(\boldsymbol{y}, \boldsymbol{x}, \boldsymbol{\theta})}{f(\boldsymbol{x}, \boldsymbol{\theta})}\frac{f(\boldsymbol{x}, \boldsymbol{\theta})}{f(\boldsymbol{\theta})} \\ &= f(\boldsymbol{y}|\boldsymbol{x}, \boldsymbol{\theta}) f(\boldsymbol{x}|\boldsymbol{\theta}) \end{aligned} \tag{10}$$

The Markovian structure of our model allows for the following decompositions:

$$f(\boldsymbol{y}|\boldsymbol{x}, \boldsymbol{\theta}) = \prod_{t=1}^{T} f(\boldsymbol{y}_t|\boldsymbol{x}_t, \boldsymbol{\theta}) \tag{11}$$

$$f(\boldsymbol{x}|\boldsymbol{\theta}) = f(\boldsymbol{x}_1)\prod_{t=2}^{T} f(\boldsymbol{x}_t|\boldsymbol{x}_{t-1}, \boldsymbol{\theta}) \tag{12}$$

where we use T rather than T_k for notational brevity. After substitution we have:

$$\begin{aligned} \log(f(\boldsymbol{y}, \boldsymbol{x}|\boldsymbol{\theta})) &= \log\left(\prod_{t=1}^{T}\left[f(\boldsymbol{y}_t|\boldsymbol{x}_t, \boldsymbol{\theta})\right] f(\boldsymbol{x}_1)\prod_{t=2}^{T}\left[f(\boldsymbol{x}_t|\boldsymbol{x}_{t-1}, \boldsymbol{\theta})\right]\right) \\ &= \sum_{t=1}^{T}\log(f(\boldsymbol{y}_t|\boldsymbol{x}_t, \boldsymbol{\theta})) + \log(f(\boldsymbol{x}_1)) + \sum_{t=2}^{T}\log(f(\boldsymbol{x}_t|\boldsymbol{x}_{t-1}, \boldsymbol{\theta})) \end{aligned} \tag{13}$$

Using the state space model, we have expressions for these conditional probabilities:

$$\begin{aligned} \log(f(\boldsymbol{y}_t|\boldsymbol{x}_t, \boldsymbol{\theta})) = &-\frac{n}{2}\log(2\pi) - \frac{1}{2}\log(|\mathbf{R}|) \\ &- \frac{1}{2}\left[\boldsymbol{y}_t^\top\mathbf{R}^{-1}\boldsymbol{y}_t - \boldsymbol{y}_t^\top\mathbf{R}^{-1}\mathbf{A}\boldsymbol{x}_t - \boldsymbol{y}_t^\top\mathbf{R}^{-1}\boldsymbol{c}\right] \\ &- \frac{1}{2}\left[-\boldsymbol{x}_t^\top\mathbf{A}^\top\mathbf{R}^{-1}\boldsymbol{y}_t + \boldsymbol{x}_t^\top\mathbf{A}^\top\mathbf{R}^{-1}\mathbf{A}\boldsymbol{x}_t + \boldsymbol{x}_t^\top\mathbf{A}^\top\mathbf{R}^{-1}\boldsymbol{c}\right] \\ &- \frac{1}{2}\left[-\boldsymbol{c}^\top\mathbf{R}^{-1}\boldsymbol{y}_t + \boldsymbol{c}^\top\mathbf{R}^{-1}\mathbf{A}\boldsymbol{x}_t + \boldsymbol{c}^\top\mathbf{R}^{-1}\boldsymbol{c}\right] \end{aligned} \tag{14}$$

$$\begin{aligned} \log(f(\boldsymbol{x}_1)) = &-\frac{m}{2}\log(2\pi) - \frac{1}{2}\log(|\boldsymbol{\Lambda}|) \\ &- \frac{1}{2}\left[\boldsymbol{x}_1^\top\boldsymbol{\Lambda}^{-1}\boldsymbol{x}_1 - \boldsymbol{x}_1^\top\boldsymbol{\Lambda}^{-1}\boldsymbol{\mu}_1 - \boldsymbol{\mu}_1^\top\boldsymbol{\Lambda}^{-1}\boldsymbol{x}_1 + \boldsymbol{\mu}_1^\top\boldsymbol{\Lambda}^{-1}\boldsymbol{\mu}_1\right] \end{aligned} \tag{15}$$

$$\begin{aligned}\log(f(\boldsymbol{x}_t|\boldsymbol{x}_{t-1},\boldsymbol{\theta})) = -\frac{m}{2}\log(2\pi) - \frac{1}{2}\log(|\mathbf{Q}|)\\ -\frac{1}{2}\left[\boldsymbol{x}_t^\top\mathbf{Q}^{-1}\boldsymbol{x}_t - \boldsymbol{x}_t^\top\mathbf{Q}^{-1}\mathbf{B}\boldsymbol{x}_{t-1} - \boldsymbol{x}_t^\top\mathbf{Q}^{-1}\boldsymbol{d}\right]\\ -\frac{1}{2}\left[-\boldsymbol{x}_{t-1}^\top\mathbf{B}^\top\mathbf{Q}^{-1}\boldsymbol{x}_t + \boldsymbol{x}_{t-1}^\top\mathbf{B}^\top\mathbf{Q}^{-1}\mathbf{B}\boldsymbol{x}_{t-1} + \boldsymbol{x}_{t-1}^\top\mathbf{B}^\top\mathbf{Q}^{-1}\boldsymbol{d}\right]\\ -\frac{1}{2}\left[-\boldsymbol{d}^\top\mathbf{Q}^{-1}\boldsymbol{x}_t + \boldsymbol{d}^\top\mathbf{Q}^{-1}\mathbf{B}\boldsymbol{x}_{t-1} + \boldsymbol{d}^\top\mathbf{Q}^{-1}\boldsymbol{d}\right]\end{aligned} \tag{16}$$

Note that these quantities are scalars, meaning each expression is equal to its transpose. The expected joint likelihood, ψ, is thus given by:

$$\begin{aligned}\psi = \mathbb{E}_{x\sim Q}\left[\log(f(\boldsymbol{y},\boldsymbol{x}|\boldsymbol{\theta})\right] = \mathbb{E}_{x\sim Q}\Bigg[\sum_{t=1}^{T}\Bigg(-\frac{n}{2}\log(2\pi) - \frac{1}{2}\log(|\mathbf{R}|)\\ -\frac{1}{2}\left[\boldsymbol{y}_t^\top\mathbf{R}^{-1}\boldsymbol{y}_t + \boldsymbol{x}_t^\top\mathbf{A}^\top\mathbf{R}^{-1}\mathbf{A}\boldsymbol{x}_t + \boldsymbol{c}^\top\mathbf{R}^{-1}\boldsymbol{c}\right]\\ -\frac{1}{2}\left[-2\boldsymbol{y}_t^\top\mathbf{R}^{-1}\mathbf{A}\boldsymbol{x}_t - 2\boldsymbol{y}_t^\top\mathbf{R}^{-1}\boldsymbol{c} + 2\boldsymbol{x}_t^\top\mathbf{A}^\top\mathbf{R}^{-1}\boldsymbol{c}\right]\Bigg)\\ + -\frac{m}{2}\log(2\pi) - \frac{1}{2}\log(|\boldsymbol{\Lambda}|)\\ -\frac{1}{2}\left[\boldsymbol{x}_1^\top\boldsymbol{\Lambda}^{-1}\boldsymbol{x}_1 - 2\boldsymbol{x}_1^\top\boldsymbol{\Lambda}^{-1}\boldsymbol{\mu}_1 + \boldsymbol{\mu}_1^\top\boldsymbol{\Lambda}^{-1}\boldsymbol{\mu}_1\right]\\ + \sum_{t=2}^{T}\Bigg(-\frac{m}{2}\log(2\pi) - \frac{1}{2}\log(|\mathbf{Q}|)\\ -\frac{1}{2}\left[\boldsymbol{x}_t^\top\mathbf{Q}^{-1}\boldsymbol{x}_t + \boldsymbol{x}_{t-1}^\top\mathbf{B}^\top\mathbf{Q}^{-1}\mathbf{B}\boldsymbol{x}_{t-1} + \boldsymbol{d}^\top\mathbf{Q}^{-1}\boldsymbol{d}\right]\\ -\frac{1}{2}\left[-2\boldsymbol{x}_{t-1}^\top\mathbf{B}^\top\mathbf{Q}^{-1}\boldsymbol{x}_t - 2\boldsymbol{d}^\top\mathbf{Q}^{-1}\boldsymbol{x}_t + 2\boldsymbol{d}^\top\mathbf{Q}^{-1}\mathbf{B}\boldsymbol{x}_{t-1}\right]\Bigg)\Bigg]\end{aligned} \tag{17}$$

Where $Q(\boldsymbol{x}) = f(\boldsymbol{x}|\boldsymbol{y},\boldsymbol{\theta})$. We can calculate closed-form parameter updates for the M-Step by differentiating with respect to each parameter, setting the resulting expression to zero, and solving. For brevity, we drop the expectation subscript. We follow a similar presentation to that given in Holmes [9], including the calculation of expected values (see Section 2.2). Note that we follow the denominator layout for vector and matrix derivatives. For matrix derivatives we refer to Petersen and Pedersen [16].

S3.3.1 A update

The $\mathbf{A}$ matrix is fully estimated. We have:

$$\begin{aligned}\frac{\partial\psi}{\partial\mathbf{A}} &= \frac{\partial}{\partial\mathbf{A}}\left[-\frac{1}{2}\sum_{t=1}^{T}\mathbb{E}\left[\boldsymbol{x}_t^\top\mathbf{A}^\top\mathbf{R}^{-1}\mathbf{A}\boldsymbol{x}_t - 2\boldsymbol{y}_t^\top\mathbf{R}^{-1}\mathbf{A}\boldsymbol{x}_t + 2\boldsymbol{x}_t^\top\mathbf{A}^\top\mathbf{R}^{-1}\boldsymbol{c}\right]\right]\\ &= -\frac{1}{2}\sum_{t=1}^{T}2\mathbf{R}^{-1}\mathbf{A}\mathbb{E}[\boldsymbol{x}_t\boldsymbol{x}_t^\top] - 2\mathbf{R}^{-1}\mathbb{E}[\boldsymbol{y}_t\boldsymbol{x}_t^\top] + 2\mathbf{R}^{-1}\boldsymbol{c}\mathbb{E}[\boldsymbol{x}_t^\top]\end{aligned} \tag{18}$$

Setting to zero and solving gives:

$$\mathbf{A} = \left(\sum_{t=1}^{T}\mathbb{E}\left[\boldsymbol{y}_t\boldsymbol{x}_t^\top\right] - \boldsymbol{c}\mathbb{E}\left[\boldsymbol{x}_t^\top\right]\right)\left(\sum_{t=1}^{T}\mathbb{E}\left[\boldsymbol{x}_t\boldsymbol{x}_t^T\right]\right)^{-1} \tag{19}$$

S3.3.2 B update

Only the diagonal of the $\mathbf{B}$ matrix is estimated. The complete matrix derivative can be used for the update rule (here we avoid converting to vectorized notation and instead note that off-diagonal entries must be

zeroed after applying the partial derivative). We have:

$$\begin{aligned}\frac{\partial \psi}{\partial \mathbf{B}} &= \frac{\partial}{\partial \mathbf{B}}\left[-\frac{1}{2}\sum_{t=2}^{T}\mathbb{E}\left[\boldsymbol{x}_{t-1}^\top \mathbf{B}^\top \mathbf{Q}^{-1}\mathbf{B}\boldsymbol{x}_{t-1} - 2\boldsymbol{x}_{t-1}^\top \mathbf{B}^\top \mathbf{Q}^{-1}\boldsymbol{x}_t + 2\boldsymbol{d}^\top \mathbf{Q}^{-1}\mathbf{B}\boldsymbol{x}_{t-1}\right]\right] \\ &= -\frac{1}{2}\sum_{t=2}^{T}\left(2\mathbf{Q}^{-1}\mathbf{B}\mathbb{E}\left[\boldsymbol{x}_{t-1}\boldsymbol{x}_{t-1}^\top\right] - 2\mathbb{E}\left[\mathbf{Q}^{-1}\boldsymbol{x}_t\boldsymbol{x}_{t-1}^\top\right] + 2\mathbb{E}\left[\mathbf{Q}^{-1}\boldsymbol{d}\boldsymbol{x}_{t-1}^\top\right]\right)\end{aligned} \tag{20}$$

Setting to zero and solving gives:

$$\mathbf{B} = \left(\sum_{t=2}^{T}\mathbb{E}\left[\boldsymbol{x}_t\boldsymbol{x}_{t-1}^\top\right] - \boldsymbol{d}\mathbb{E}\left[\boldsymbol{x}_{t-1}^\top\right]\right)\left(\sum_{t=2}^{T}\mathbb{E}\left[\boldsymbol{x}_{t-1}\boldsymbol{x}_{t-1}^T\right]\right)^{-1} \tag{21}$$

S3.3.3 c update

The entire $\boldsymbol{c}$ vector is estimated. We have:

$$\begin{aligned}\frac{\partial \psi}{\partial \boldsymbol{c}} &= \frac{\partial}{\partial \mathbf{c}}\left[-\frac{1}{2}\sum_{t=1}^{T}\mathbb{E}\left[\boldsymbol{c}^\top \mathbf{R}^{-1}\boldsymbol{c} - 2\boldsymbol{y}_t^\top \mathbf{R}^{-1}\boldsymbol{c} + 2\boldsymbol{x}_t^\top \mathbf{A}^\top \mathbf{R}^{-1}\boldsymbol{c}\right]\right] \\ &= -\frac{1}{2}\sum_{t=1}^{T}2\mathbb{E}\left[\mathbf{R}^{-1}\boldsymbol{c} - 2\mathbf{R}^{-1}\boldsymbol{y}_t + 2\mathbf{R}^{-1}\mathbf{A}\boldsymbol{x}_t\right]\end{aligned} \tag{22}$$

Setting to zero and solving gives:

$$\boldsymbol{c} = \frac{1}{T}\sum_{t=1}^{T}\left(\mathbb{E}\left[\boldsymbol{y}_t\right] - \mathbf{A}\mathbb{E}\left[\boldsymbol{x}_t\right]\right) \tag{23}$$

S3.3.4 d update

The entire $\boldsymbol{d}$ vector is estimated. We have:

$$\begin{aligned}\frac{\partial \psi}{\partial \boldsymbol{d}} &= \frac{\partial}{\partial \boldsymbol{d}}\left[-\frac{1}{2}\sum_{t=2}^{T}\mathbb{E}\left[\boldsymbol{d}^\top \mathbf{Q}^{-1}\boldsymbol{d} - 2\boldsymbol{d}^\top \mathbf{Q}^{-1}\boldsymbol{x}_t + 2\boldsymbol{d}^\top \mathbf{Q}^{-1}\mathbf{B}\boldsymbol{x}_{t-1}\right]\right] \\ &= -\frac{1}{2}\sum_{t=2}^{T}\mathbb{E}\left[2\mathbf{Q}^{-1}\boldsymbol{d} - 2\mathbf{Q}^{-1}\boldsymbol{x}_t + 2\mathbf{Q}^{-1}\mathbf{B}\boldsymbol{x}_{t-1}\right]\end{aligned} \tag{24}$$

Setting to zero and solving gives:

$$\boldsymbol{d} = \frac{1}{T-1}\sum_{t=2}^{T}\left(\mathbb{E}\left[\boldsymbol{x}_t\right] - \mathbf{B}\mathbb{E}\left[\boldsymbol{x}_{t-1}\right]\right) \tag{25}$$

S3.3.5 R update

Only the diagonal of the $\mathbf{R}$ matrix is estimated. The complete matrix derivative can be used for the update rule (here we avoid converting to vectorized notation and instead note that off-diagonal entries must be zeroed after applying the partial derivative). We have:

$$\begin{aligned}\frac{\partial \psi}{\partial \mathbf{R}} &= \frac{\partial}{\partial \mathbf{R}}\Big[-\frac{1}{2}\sum_{t=1}^{T}\mathbb{E}\big[\log(|\mathbf{R}|) + \boldsymbol{y}_t^\top \mathbf{R}^{-1}\boldsymbol{y}_t + \boldsymbol{x}_t^\top \mathbf{A}^\top \mathbf{R}^{-1}\mathbf{A}\boldsymbol{x}_t + \boldsymbol{c}^\top \mathbf{R}^{-1}\boldsymbol{c} \\ &\qquad - 2\boldsymbol{y}_t^\top \mathbf{R}^{-1}\mathbf{A}\boldsymbol{x}_t - 2\boldsymbol{y}_t^\top \mathbf{R}^{-1}\boldsymbol{c} + 2\boldsymbol{x}_t^\top \mathbf{A}^\top \mathbf{R}^{-1}\boldsymbol{c}\big]\Big] \\ &= -\frac{1}{2}\sum_{t=1}^{T}\mathbb{E}\big[\mathbf{R}^{-1} + \mathbf{R}^{-1}(-\boldsymbol{y}_t\boldsymbol{y}_t^\top - \mathbf{A}\boldsymbol{x}_t\boldsymbol{x}_t^\top \mathbf{A}^\top - \boldsymbol{c}\boldsymbol{c}^\top + 2\boldsymbol{y}_t\boldsymbol{x}_t^\top \mathbf{A}^\top \\ &\qquad + 2\boldsymbol{y}_t\boldsymbol{c}^\top - 2\mathbf{A}\boldsymbol{x}_t\boldsymbol{c}^\top)\mathbf{R}^{-1}\big]\end{aligned} \tag{26}$$

Setting to zero and solving gives:

$$\mathbf{R} = \frac{1}{T}\sum_{t=1}^{T} \mathbb{E}\left[\boldsymbol{y}_t\boldsymbol{y}_t^\top\right] + \mathbf{A}\mathbb{E}\left[\boldsymbol{x}_t\boldsymbol{x}_t^\top\right]\mathbf{A}^\top + \boldsymbol{c}\boldsymbol{c}^\top - 2\mathbb{E}\left[\boldsymbol{y}_t\boldsymbol{x}_t^\top\right]\mathbf{A}^\top - 2\mathbb{E}\left[\boldsymbol{y}_t\right]\boldsymbol{c}^\top + 2\mathbf{A}\mathbb{E}\left[\boldsymbol{x}_t\right]\boldsymbol{c}^\top \tag{27}$$

S3.4 Maximum a posteriori estimation

In the case of MAP estimation, we wish to find the model parameters $\boldsymbol{\theta}$ that maximize the posterior probability for each individual, defined as:

$$f(\boldsymbol{\theta}|\boldsymbol{y}) = \frac{f(\boldsymbol{\theta})f(\boldsymbol{y}|\boldsymbol{\theta})}{f(\boldsymbol{y})} = \frac{f(\boldsymbol{\theta})\int_{\mathbf{X}} f(\boldsymbol{y},\boldsymbol{x}|\boldsymbol{\theta})d\mathbf{x}}{f(\boldsymbol{y})}$$

As before, we can find the optimal $\boldsymbol{\theta}$ by instead maximizing the log of the posterior. We similarly introduce an arbitrary distribution $Q(\boldsymbol{x})$ over the latent states and apply Jensen's inequality:

$$\begin{aligned}
\log(f(\boldsymbol{\theta}|\boldsymbol{y})) &= \log(f(\boldsymbol{\theta})) + \log\left(\int_{\mathbf{X}} f(\boldsymbol{y},\boldsymbol{x}|\boldsymbol{\theta})d\mathbf{x}\right) - \log(f(\boldsymbol{y})) \\
&= \log(f(\boldsymbol{\theta})) + \log\left(\mathbb{E}_{x\sim Q}\left[\frac{f(\boldsymbol{y},\boldsymbol{x}|\boldsymbol{\theta})}{Q(\boldsymbol{x})}\right]\right) - \log(f(\boldsymbol{y})) \\
&\geq \log(f(\boldsymbol{\theta})) + \mathbb{E}_{x\sim Q}\left[\log\left(\frac{f(\boldsymbol{y},\boldsymbol{x}|\boldsymbol{\theta})}{Q(\boldsymbol{x})}\right)\right] - \log(f(\boldsymbol{y})) \\
&= \log(f(\boldsymbol{\theta})) + \mathbb{E}_{x\sim Q}\left[\log(f(\boldsymbol{y},\boldsymbol{x}|\boldsymbol{\theta})\right] - \mathbb{E}_{x\sim Q}\left[\log(Q(\boldsymbol{x})\right] - \log(f(\boldsymbol{y}))
\end{aligned} \tag{28}$$

Since $f(\boldsymbol{\theta})$ and $f(\boldsymbol{y})$ do not depend on $Q(\boldsymbol{x})$, we make the inequality tight with the choice $Q(\boldsymbol{x}) = f(\boldsymbol{x}|\boldsymbol{y},\boldsymbol{\theta})$. Next we consider the terms derived from the prior, noting that we assume independent prior distributions for each estimated parameter:

$$\phi = \log\left(f(\boldsymbol{\theta})\right) = \sum_{i=1}^{n}\sum_{j=1}^{n}\phi_{a_{ij}} + \sum_{i=1}^{n}\phi_{c_i} + \sum_{i=1}^{m}\phi_{b_{ii}} + \sum_{i=1}^{m}\phi_{d_i} + \sum_{i=1}^{n-1}\phi_{r_{ii}} \tag{29}$$

where $\phi_{\text{parameter}}$ denotes the log-density for that parameter. Recalling our definition of ψ in Eqn. 17 we apply a similar EM procedure, maximizing only the terms which depend on $\boldsymbol{\theta}$ during the M-Step:

Algorithm 2 Expectation-Maximization (MAP)

Input : Initial parameter iterate $\boldsymbol{\theta}_0$, prior distribution $f(\boldsymbol{\theta})$, observed data series $\boldsymbol{y}$

$k \leftarrow 0$
repeat
  (E-Step) $Q_{k+1}(\boldsymbol{x}) \leftarrow f(\boldsymbol{x}|\boldsymbol{y},\boldsymbol{\theta}_k)$
  (M-Step) $\boldsymbol{\theta}_{k+1} \leftarrow \underset{\boldsymbol{\theta}}{\operatorname{argmax}}\ \psi_{Q_{k+1}}(\boldsymbol{\theta}) + \phi(\boldsymbol{\theta})$
  $k \leftarrow k+1$
until converged

We can calculate closed-form parameter updates for the M-Step by differentiating $\psi + \phi$ with respect to each parameter, setting the resulting expression to zero, and solving (the particular prior distributions for each parameter were chosen to allow for such a closed form solution).

S3.4.1 A update

The $\mathbf{A}$ matrix is fully estimated. We have:

$$\begin{aligned}\frac{\partial\psi}{\partial\mathbf{A}} &= \frac{\partial}{\partial\mathbf{A}}\left[-\frac{1}{2}\sum_{t=1}^{T}\mathbb{E}\left[\boldsymbol{x}_t^\top\mathbf{A}^\top\mathbf{R}^{-1}\mathbf{A}\boldsymbol{x}_t - 2\boldsymbol{y}_t^\top\mathbf{R}^{-1}\mathbf{A}\boldsymbol{x}_t + 2\boldsymbol{x}_t^\top\mathbf{A}^\top\mathbf{R}^{-1}\boldsymbol{c}\right]\right] \\ &= -\frac{1}{2}\sum_{t=1}^{T}2\mathbf{R}^{-1}\mathbf{A}\mathbb{E}[\boldsymbol{x}_t\boldsymbol{x}_t^\top] - 2\mathbf{R}^{-1}\mathbb{E}[\boldsymbol{y}_t\boldsymbol{x}_t^\top] + 2\mathbf{R}^{-1}\boldsymbol{c}\mathbb{E}[\boldsymbol{x}_t^\top]\end{aligned} \tag{30}$$

We use an independent Gaussian prior for each $a_{ij} \in \mathbf{A}$ with $a_{ij} \sim \mathcal{N}(\mu_{a_{ij}}, \sigma^2_{a_{ij}})$. This results in a corresponding addition to the log likelihood of ϕ_A:

$$\phi_A = \sum_{i=1}^{n}\sum_{j=1}^{m} -\frac{1}{2}\log(2\pi) - \frac{1}{2}\log(\sigma^2_{a_{ij}}) - \frac{1}{2}\frac{1}{\sigma^2_{a_{ij}}}(a_{ij} - \mu_{a_{ij}})^2 \tag{31}$$

Note that this gives us:

$$\frac{\partial\phi_A}{\partial a_{ij}} = -\frac{1}{\sigma^2_{a_{ij}}}(a_{ij} - \mu_{a_{ij}}) \tag{32}$$

To find the M-Step update for $\mathbf{A}$, we take the derivative of $\psi + \phi$ with respect to $\mathbf{A}$, set the expression to 0, and solve for $\mathbf{A}$:

$$\frac{\partial(\psi+\phi)}{\partial\mathbf{A}} = -\frac{1}{2}\sum_{t=1}^{T}\left(2\mathbf{R}^{-1}\mathbf{A}\mathbb{E}[\boldsymbol{x}_t\boldsymbol{x}_t^\top] - 2\mathbf{R}^{-1}\mathbb{E}[\boldsymbol{y}_t\boldsymbol{x}_t^\top] + 2\mathbf{R}^{-1}\boldsymbol{c}\mathbb{E}[\boldsymbol{x}_t^\top]\right) - \tilde{\mathbf{\Sigma}}_A \circ \mathbf{A} + \tilde{\mathbf{\Sigma}}_A \circ \tilde{\mu}_A \tag{33}$$

Where we define $\tilde{\Sigma}_A$ to be the $n \times m$ matrix with entry (i, j) equal to $\frac{1}{\sigma^2_{a_{ij}}}$ and $\tilde{\mu}_A$ is the $n \times m$ matrix with entry (i, j) equal to $\mu_{a_{ij}}$. Setting to 0 and solving gives:

$$\mathbf{A}\left(\sum_{t=1}^{T}\mathbb{E}\left[\boldsymbol{x}_t\boldsymbol{x}_t^\top\right]\right) + \mathbf{A} \circ \mathbf{R}\tilde{\mathbf{\Sigma}}_A = \sum_{t=1}^{T}\left(\mathbb{E}\left[\boldsymbol{y}_t\boldsymbol{x}_t^\top\right] - \boldsymbol{c}\mathbb{E}\left[\boldsymbol{x}_t\right]^\top\right) + \mathbf{R}\left(\tilde{\mathbf{\Sigma}}_A \circ \tilde{\mu}_A\right) \tag{34}$$

Here we used the helpful property that $\mathbf{R}(\tilde{\mathbf{\Sigma}}_A \circ \mathbf{A}) = \mathbf{A} \circ \mathbf{R}\tilde{\mathbf{\Sigma}}_A$ when $\mathbf{R}$ is diagonal (assumed in our model) [10]. Note that a system of the form $\mathbf{AC} + \mathbf{A} \circ \mathbf{D} = \mathbf{E}$ can be solved for $\mathbf{A}$ row-by-row. Each row of $\mathbf{A}$, denoted $\boldsymbol{a}_k$ $k = 1, \ldots, n$, is given by $\boldsymbol{a}_k = \boldsymbol{e}_k(\mathbf{C} + \mathrm{Diag}(\boldsymbol{d}_k))^+$, where $\boldsymbol{d}_k$ and $\boldsymbol{e}_k$ are the kth rows of $\mathbf{D}$ and $\mathbf{E}$, respectively, and $^+$ denotes the pseudoinverse.

S3.4.2 B update

Only the diagonal of the $\mathbf{B}$ matrix is estimated. The complete matrix derivative can be used to derive the update rule (here we avoid converting to vectorized notation and instead note that off-diagonal entries must be zeroed after applying the partial derivative). We have:

$$\begin{aligned}\frac{\partial\psi}{\partial\mathbf{B}} &= \frac{\partial}{\partial\mathbf{B}}\left[-\frac{1}{2}\sum_{t=2}^{T}\mathbb{E}\left[\boldsymbol{x}_{t-1}^\top\mathbf{B}^\top\mathbf{Q}^{-1}\mathbf{B}\boldsymbol{x}_{t-1} - 2\boldsymbol{x}_{t-1}^\top\mathbf{B}^\top\mathbf{Q}^{-1}\boldsymbol{x}_t + 2\boldsymbol{d}^\top\mathbf{Q}^{-1}\mathbf{B}\boldsymbol{x}_{t-1}\right]\right] \\ &= -\frac{1}{2}\sum_{t=2}^{T}\left(2\mathbf{Q}^{-1}\mathbf{B}\mathbb{E}\left[\boldsymbol{x}_{t-1}\boldsymbol{x}_{t-1}^\top\right] - 2\mathbb{E}\left[\mathbf{Q}^{-1}\boldsymbol{x}_t\boldsymbol{x}_{t-1}^\top\right] + 2\mathbb{E}\left[\mathbf{Q}^{-1}\boldsymbol{d}\boldsymbol{x}_{t-1}^\top\right]\right)\end{aligned} \tag{35}$$

We use an independent truncated Gaussian prior for each $b_{ii} \in \mathbf{B}$, $i = 1, \ldots, m$, with $b_{ii} \sim \mathcal{TN}(\mu_{b_{ii}}, \sigma^2_{b_{ii}}, \bar{a}, \bar{b})$, i.e. the density is defined only between $\bar{a}$ and $\bar{b}$, chosen to be -1 and 1 to enforce model stability. This

results in a corresponding addition to the log likelihood of ϕ_B:

$$\begin{aligned}\phi_B = -\infty \cdot \mathbf{1}_{\{b \notin [\bar{a}, \bar{b}]\}} + \sum_{i=1}^{m} -\frac{1}{2}\log(2\pi) - \frac{1}{2}\log(\sigma_{b_{ii}}^2) - \frac{1}{2}\frac{1}{\sigma_{b_{ii}}^2}(b_{ii} - \mu_{b_{ii}})^2 \\ - \log\left(\Phi\left(\frac{\bar{b} - \mu_{b_{ii}}}{\sigma_{b_{ii}}}\right) - \Phi\left(\frac{-\bar{a} - \mu_{b_{ii}}}{\sigma_{b_{ii}}}\right)\right)\end{aligned} \tag{36}$$

The indicator effectively restricts the optimization to values for b_{ii} within $[\bar{a}, \bar{b}]$. Noting this restriction we have:

$$\frac{\partial \phi_B}{\partial b_{ii}} = -\frac{1}{\sigma_{b_{ii}}^2}(b_{ii} - \mu_{b_{ii}}) \tag{37}$$

To find the M-Step update for $\mathbf{B}$, we take the derivative of $\psi + \phi$ with respect to $\mathbf{B}$, set the expression to 0, and solve for $\mathbf{B}$. Again, note that off-diagonal values are zeroed following the evaluation of the partial derivatives.

$$\begin{aligned}\frac{\partial(\psi + \phi)}{\partial \mathbf{B}} &= -\frac{1}{2}\sum_{t=2}^{T}\left(2\mathbf{Q}^{-1}\mathbf{B}\mathbb{E}\left[\boldsymbol{x}_{t-1}\boldsymbol{x}_{t-1}^\top\right] - 2\mathbf{Q}^{-1}\mathbb{E}\left[\boldsymbol{x}_t\boldsymbol{x}_{t-1}^\top\right] + 2\mathbf{Q}^{-1}\boldsymbol{d}\mathbb{E}\left[\boldsymbol{x}_{t-1}^\top\right]\right) \\ &\qquad - \tilde{\mathbf{\Sigma}}_B \circ \mathbf{B} + \tilde{\mathbf{\Sigma}}_B \circ \tilde{\mu}_B \\ &= -\sum_{t=2}^{T}\left(\mathbf{Q}^{-1}\mathbf{B}\mathbb{E}\left[\boldsymbol{x}_{t-1}\boldsymbol{x}_{t-1}^\top\right] - \mathbf{Q}^{-1}\mathbb{E}\left[\boldsymbol{x}_t\boldsymbol{x}_{t-1}^\top\right] + \mathbf{Q}^{-1}\boldsymbol{d}\mathbb{E}\left[\boldsymbol{x}_{t-1}^\top\right]\right) \\ &\qquad - \mathbf{B}\tilde{\mathbf{\Sigma}}_B + \tilde{\mathbf{\Sigma}}_B\tilde{\mu}_B\end{aligned} \tag{38}$$

Where we define $\tilde{\mathbf{\Sigma}}_B$ to be the $m \times m$ matrix with entries (i, i) equal to $\frac{1}{\sigma_{b_{ii}}^2}$, $i = 1, \ldots, m$, and 0 otherwise. We similarly define $\tilde{\mu}_B$ to be the $m \times m$ matrix with entries (i, i) equal to $\mu_{b_{ii}}$ and 0 otherwise. Note that for diagonal matrices, the Hadamard product reduces to typical matrix multiplication. Setting to 0 and solving (recalling that we assume $\mathbf{Q} = \mathbf{I}_m$ in our modeling) gives:

$$\mathbf{B} = \left(\tilde{\mathbf{\Sigma}}_B\tilde{\mu}_B + \sum_{t=2}^{T}\left(\mathbb{E}\left[\boldsymbol{x}_t\boldsymbol{x}_{t-1}^\top\right] - \boldsymbol{d}\mathbb{E}\left[\boldsymbol{x}_{t-1}^\top\right]\right)\right)\left(\tilde{\mathbf{\Sigma}}_B + \sum_{t=2}^{T}\mathbb{E}\left[\boldsymbol{x}_{t-1}\boldsymbol{x}_{t-1}^\top\right]\right)^+ \tag{39}$$

S3.4.3 c update

The entire $\boldsymbol{c}$ vector is estimated. We have:

$$\begin{aligned}\frac{\partial \psi}{\partial \boldsymbol{c}} &= \frac{\partial}{\partial \mathbf{c}}\left[-\frac{1}{2}\sum_{t=1}^{T}\mathbb{E}\left[\boldsymbol{c}^\top\mathbf{R}^{-1}\boldsymbol{c} - 2\boldsymbol{y}_t^\top\mathbf{R}^{-1}\boldsymbol{c} + 2\boldsymbol{x}_t^\top\mathbf{A}^\top\mathbf{R}^{-1}\boldsymbol{c}\right]\right] \\ &= -\frac{1}{2}\sum_{t=1}^{T}2\mathbb{E}\left[\mathbf{R}^{-1}\boldsymbol{c} - 2\mathbf{R}^{-1}\boldsymbol{y}_t + 2\mathbf{R}^{-1}\mathbf{A}\boldsymbol{x}_t\right]\end{aligned} \tag{40}$$

We use an independent Gaussian prior for each $c_i \in \boldsymbol{c}$, $i = 1, \ldots, n$, with $c_i \sim \mathcal{N}(\mu_{c_i}, \sigma_{c_i}^2)$. This results in a corresponding addition to the log likelihood of ϕ_c:

$$\phi_c = \sum_{i=1}^{n} -\frac{1}{2}\log(2\pi) - \frac{1}{2}\log(\sigma_{c_i}^2) - \frac{1}{2}\frac{1}{\sigma_{c_i}^2}(c_i - \mu_{c_i})^2 \tag{41}$$

Note that we have:

$$\frac{\partial \phi_c}{\partial c_i} = -\frac{1}{\sigma_{c_i}^2}(c_i - \mu_{c_i}) \tag{42}$$

To find the M-Step update for $\boldsymbol{c}$, we take the derivative of $\psi + \phi$ with respect to $\boldsymbol{c}$, set the expression to 0, and solve for $\boldsymbol{c}$:

$$\frac{\partial(\psi+\phi)}{\partial \boldsymbol{c}} = -\frac{1}{2}\sum_{t=1}^{T}\left(2\mathbb{E}\left[\mathbf{R}^{-1}\boldsymbol{c} - 2\mathbf{R}^{-1}\boldsymbol{y}_t + 2\mathbf{R}^{-1}\mathbf{A}\boldsymbol{x}_t\right]\right) - \tilde{\boldsymbol{\Sigma}}_c\boldsymbol{c} + \tilde{\boldsymbol{\Sigma}}_c\tilde{\boldsymbol{\mu}}_c \tag{43}$$

Where we define $\tilde{\boldsymbol{\Sigma}}_c$ to be an $n \times n$ diagonal matrix with $\frac{1}{\sigma^2_{c_i}}$, $i = 1, \ldots, n$, along the diagonal. We define $\tilde{\boldsymbol{\mu}}_c$ to be an $n \times 1$ vector with entries μ_{c_i}, $i = 1, \ldots, n$. Setting to zero and solving yields:

$$\boldsymbol{c} = \left(T\mathbf{I}_n + \mathbf{R}\tilde{\boldsymbol{\Sigma}}_c\right)^{+}\left(\sum_{t=1}^{T}\left(\mathbb{E}\left[\boldsymbol{y}_t\right] - \mathbf{A}\mathbb{E}\left[\boldsymbol{x}_t\right]\right) + \mathbf{R}\tilde{\boldsymbol{\Sigma}}_c\tilde{\boldsymbol{\mu}}_c\right) \tag{44}$$

S3.4.4 d update

The entire $\boldsymbol{d}$ vector is estimated. We have:

$$\begin{aligned}\frac{\partial \psi}{\partial \boldsymbol{d}} &= \frac{\partial}{\partial \boldsymbol{d}}\left[-\frac{1}{2}\sum_{t=2}^{T}\mathbb{E}\left[\boldsymbol{d}^{\top}\mathbf{Q}^{-1}\boldsymbol{d} - 2\boldsymbol{d}^{\top}\mathbf{Q}^{-1}\boldsymbol{x}_t + 2\boldsymbol{d}^{\top}\mathbf{Q}^{-1}\mathbf{B}\boldsymbol{x}_{t-1}\right]\right] \\ &= -\frac{1}{2}\sum_{t=2}^{T}\mathbb{E}\left[2\mathbf{Q}^{-1}\boldsymbol{d} - 2\mathbf{Q}^{-1}\boldsymbol{x}_t + 2\mathbf{Q}^{-1}\mathbf{B}\boldsymbol{x}_{t-1}\right]\end{aligned} \tag{45}$$

We use an independent Gaussian prior for each $d_i \in \boldsymbol{d}$, $i = 1, \ldots, m$, with $d_i \sim \mathcal{N}(\mu_{d_i}, \sigma^2_{d_i})$. This results in a corresponding addition to the log likelihood of ϕ_d:

$$\phi_d = \sum_{i=1}^{m} -\frac{1}{2}\log(2\pi) - \frac{1}{2}\log(\sigma^2_{d_i}) - \frac{1}{2}\frac{1}{\sigma^2_{d_i}}(d_i - \mu_{d_i})^2 \tag{46}$$

Note that we have:

$$\frac{\partial \phi_d}{\partial d_i} = -\frac{1}{\sigma^2_{d_i}}(d_i - \mu_{d_i}) \tag{47}$$

To find the M-Step update for $\boldsymbol{d}$, we take the derivative of $\psi + \phi$ with respect to $\boldsymbol{d}$, set the expression to 0, and solve for $\boldsymbol{d}$:

$$\frac{\partial(\psi+\phi)}{\partial \boldsymbol{d}} = -\frac{1}{2}\sum_{t=2}^{T}\left(\mathbb{E}\left[2\mathbf{Q}^{-1}\boldsymbol{d} - 2\mathbf{Q}^{-1}\boldsymbol{x}_t + 2\mathbf{Q}^{-1}\mathbf{B}\boldsymbol{x}_{t-1}\right]\right) - \tilde{\boldsymbol{\Sigma}}_d\boldsymbol{d} + \tilde{\boldsymbol{\Sigma}}_d\tilde{\boldsymbol{\mu}}_d \tag{48}$$

Where we define $\tilde{\boldsymbol{\Sigma}}_d$ to be an $m \times m$ diagonal matrix with $\frac{1}{\sigma^2_{d_i}}$, $i = 1, \ldots, m$, along the diagonal. We define $\tilde{\boldsymbol{\mu}}_d$ to be an $m \times 1$ vector with entries μ_{d_i}, $i = 1, \ldots, m$. Setting to zero and solving (recalling $\mathbf{Q} = \mathbf{I}_m$ in our modeling) yields:

$$\boldsymbol{d} = \left((T-1)\mathbf{I}_m + \tilde{\boldsymbol{\Sigma}}_d\right)^{+}\left(\sum_{t=2}^{T}\left(\mathbb{E}\left[\boldsymbol{x}_t\right] - \mathbf{B}\mathbb{E}\left[\boldsymbol{x}_{t-1}\right]\right) + \tilde{\boldsymbol{\Sigma}}_d\tilde{\boldsymbol{\mu}}_d\right) \tag{49}$$

S3.4.5 R update

Only the diagonal of the $\mathbf{R}$ matrix is estimated, except for the lapse variance which is assumed to be fixed at 0.25. The complete matrix derivative can be used for the update rule (similar to the $\mathbf{B}$ case, we avoid converting to vectorized notation and instead note that off-diagonal entries must be zeroed after applying

the partial derivative). We have:

$$\begin{aligned}\frac{\partial\psi}{\partial\mathbf{R}} &= \frac{\partial}{\partial\mathbf{R}}\Big[-\frac{1}{2}\sum_{t=1}^{T}\mathbb{E}\big[\log(|\mathbf{R}|) + \boldsymbol{y}_t^\top\mathbf{R}^{-1}\boldsymbol{y}_t + \boldsymbol{x}_t^\top\mathbf{A}^\top\mathbf{R}^{-1}\mathbf{A}\boldsymbol{x}_t + \boldsymbol{c}^\top\mathbf{R}^{-1}\boldsymbol{c} \\ &\qquad - 2\boldsymbol{y}_t^\top\mathbf{R}^{-1}\mathbf{A}\boldsymbol{x}_t - 2\boldsymbol{y}_t^\top\mathbf{R}^{-1}\boldsymbol{c} + 2\boldsymbol{x}_t^\top\mathbf{A}^\top\mathbf{R}^{-1}\boldsymbol{c}\big]\Big] \\ &= -\frac{1}{2}\sum_{t=1}^{T}\mathbb{E}\big[\mathbf{R}^{-1} + \mathbf{R}^{-1}(-\boldsymbol{y}_t\boldsymbol{y}_t^\top - \mathbf{A}\boldsymbol{x}_t\boldsymbol{x}_t^\top\mathbf{A}^\top - \boldsymbol{c}\boldsymbol{c}^\top + 2\boldsymbol{y}_t\boldsymbol{x}_t^\top\mathbf{A}^\top \\ &\qquad + 2\boldsymbol{y}_t\boldsymbol{c}^\top - 2\mathbf{A}\boldsymbol{x}_t\boldsymbol{c}^\top)\mathbf{R}^{-1}\big]\end{aligned} \tag{50}$$

We use independent inverse gamma priors for the diagonal elements of $\mathbf{R}$, i.e., $r_{ii} \sim \text{IG}(\alpha_{r_{ii}}, \beta_{r_{ii}})$, $i = 1, \ldots, n-1$. This results in a corresponding addition to the log likelihood of ϕ_R:

$$\phi_R = \sum_{i=1}^{n-1}\alpha_{r_{ii}}\log(\beta_{r_{ii}}) - \log(\Gamma(\alpha_{r_{ii}})) - (\alpha_{r_{ii}}+1)\log(r_{ii}) - \frac{\beta_{r_{ii}}}{r_{ii}} \tag{51}$$

Note that we have:

$$\frac{\partial\phi_R}{\partial r_{ii}} = -(\alpha_{r_{ii}}+1)\frac{1}{r_{ii}} + \frac{\beta_{r_{ii}}}{r_{ii}^2} \tag{52}$$

To find the M-Step update for $\mathbf{R}$, we take the derivative of $\psi + \phi$ with respect to $\mathbf{R}$, set the expression to zero, and solve for $\mathbf{R}$. We show the complete matrix derivative for $\mathbf{R}$, but use only the diagonal elements for the update:

$$\begin{aligned}\frac{\partial(\psi+\phi)}{\partial\mathbf{R}} &= -\frac{1}{2}\sum_{t=1}^{T}\Big(\mathbb{E}\big[\mathbf{R}^{-1} + \mathbf{R}^{-1}(-\boldsymbol{y}_t\boldsymbol{y}_t^\top - \mathbf{A}\boldsymbol{x}_t\boldsymbol{x}_t^\top\mathbf{A}^\top - \boldsymbol{c}\boldsymbol{c}^\top + 2\boldsymbol{y}_t\boldsymbol{x}_t^\top\mathbf{A}^\top \\ &\qquad + 2\boldsymbol{y}_t\boldsymbol{c}^\top - 2\mathbf{A}\boldsymbol{x}_t\boldsymbol{c}^\top)\mathbf{R}^{-1}\big]\Big) - \tilde{\boldsymbol{\alpha}}_R \circ \mathbf{R}^{-1} + \tilde{\boldsymbol{\beta}}_R \circ \left(\mathbf{R}^{-1}\mathbf{R}^{-1}\right) \\ &= -\frac{1}{2}\sum_{t=1}^{T}\Big(\mathbf{R}^{-1} + \mathbf{R}^{-1}(-\mathbb{E}\left[\boldsymbol{y}_t\boldsymbol{y}_t^\top\right] - \mathbf{A}\mathbb{E}\left[\boldsymbol{x}_t\boldsymbol{x}_t^\top\right]\mathbf{A}^\top - \boldsymbol{c}\boldsymbol{c}^\top + 2\mathbb{E}\left[\boldsymbol{y}_t\boldsymbol{x}_t^\top\right]\mathbf{A}^\top \\ &\qquad + 2\mathbb{E}\left[\boldsymbol{y}_t\right]\boldsymbol{c}^\top - 2\mathbf{A}\mathbb{E}\left[\boldsymbol{x}_t\right]\boldsymbol{c}^\top)\mathbf{R}^{-1}\Big) - \mathbf{R}^{-1}\tilde{\boldsymbol{\alpha}}_R + \mathbf{R}^{-1}\tilde{\boldsymbol{\beta}}_R\mathbf{R}^{-1}\end{aligned} \tag{53}$$

Where $\tilde{\boldsymbol{\alpha}}_R$ is an $n \times n$ diagonal matrix with entries $\tilde{\alpha}_{ii} = (\alpha_{r_{ii}} + 1)$, $i = 1, \ldots, n$. We similarly define $\tilde{\boldsymbol{\beta}}_R$ to be an $n \times n$ diagonal matrix with entries $\beta_{ii} = \beta_{r_{ii}}$, $i = 1, \ldots, n$. Setting to zero and solving, we have:

$$\begin{aligned}\mathbf{R} &= (T\mathbf{I}_n + 2\tilde{\boldsymbol{\alpha}}_R)^+ \\ &\quad \cdot\Big(\sum_{t=1}^{T}\left(\mathbb{E}\left[\boldsymbol{y}_t\boldsymbol{y}_t^\top\right] + \mathbf{A}\mathbb{E}\left[\boldsymbol{x}_t\boldsymbol{x}_t^\top\right]\mathbf{A}^\top + \boldsymbol{c}\boldsymbol{c}^\top - 2\mathbb{E}\left[\boldsymbol{y}_t\boldsymbol{x}_t^\top\right]\mathbf{A}^\top \right. \\ &\qquad \left. - 2\mathbb{E}\left[\boldsymbol{y}_t\right]\boldsymbol{c}^\top + 2\mathbf{A}\mathbb{E}\left[\boldsymbol{x}_t\right]\boldsymbol{c}^\top\right) + 2\tilde{\boldsymbol{\beta}}_R\Big)\end{aligned} \tag{54}$$

S3.5 Generating lapse predictions

Note that a single fitted SSM can be used for multiple prediction tasks. This manuscript used Monte Carlo simulation to assess lapse risk for the different prediction tasks (i.e., same-day lapse, lapse within 3 days, lapse within 7 days). The Kalman filtering process produces a Gaussian state estimate at the current time step t, i.e., $x_t \sim \mathcal{N}(\mu, \Sigma)$. Note that the hidden state can be simulated per the estimated transition equation:

$$\boldsymbol{x}_{t+1} = \mathbf{B}\boldsymbol{x}_t + \boldsymbol{d} + \boldsymbol{w}_t \tag{55}$$

In this manuscript, $n = 20,000$ samples were drawn from the filtered state distribution and simulated forward up to 7 days to generate 20,000 corresponding state sequences of the form $(x_t, x_{t+1}, \ldots, x_{t+7})$. Each latent state value can be passed through the nth row of the observation equation to generate a lapse prediction value, $\hat{e}$ (in this modeling, the tenth row corresponds to lapse). Note that lapse is a binary outcome, but the linear-Gaussian framework of the current modeling does not restrict observation outputs to $[0, 1]$. As such, these lapse predictions are clipped to ϵ and $1 - \epsilon$, with $\epsilon = 1 \times 10^{-12}$. Because of this clipping, we chose to generate $\hat{e}$ values without noise (i.e., $\hat{e} = A_{10}x + c_{10}$), though we plan to re-address this choice in future work. Given a corresponding set of lapse predictions for the ith simulated trajectory, $(\hat{e}_t, \hat{e}_{t+1}, \ldots, \hat{e}_{t+7})_i$, the Monte Carlo estimate for the probability of lapse occurring within a given window length L is then $\frac{1}{n}\sum_{i=1}^{n}\left(1 - \prod_{k=t}^{t+L}(1 - \hat{e}_k)\right)$.

S3.6 State space model prior distributions

The prior distributions used for the state space model parameters were constructed using the following procedure. First, we fitted state space models for a population of individuals (i.e., those in the training folds) using maximum likelihood estimation. These fits provided a distribution of parameter estimates (one for each parameter and individual). We chose distributions that respected the support of each parameter, appeared to visually match the structure of the parameter estimates, and mathematically admitted closed form update rules for the MAP fitting process (see Section S3.4). The priors were calculated using the packages `MASS` and `fitdistrplus` [20, 4].

We show a few representative plots illustrating the MLE fitted parameter values across the complete population. Figure 2 shows the joint distributions of the first four rows of **A**, fitted with independent normal distributions. Figure 3 shows the distributions for the first four elements of $\boldsymbol{c}$, fitted with independent normal distributions. Figure 4 shows the distribution for the diagonal elements of **B**, fitted with truncated normal distributions (truncated between -1 and 1 to ensure system stability) and the elements of $\boldsymbol{d}$, fitted with normal distributions. Figure 5 shows the distributions for the first four diagonal elements of **R**, fitted with inverse gamma distributions.

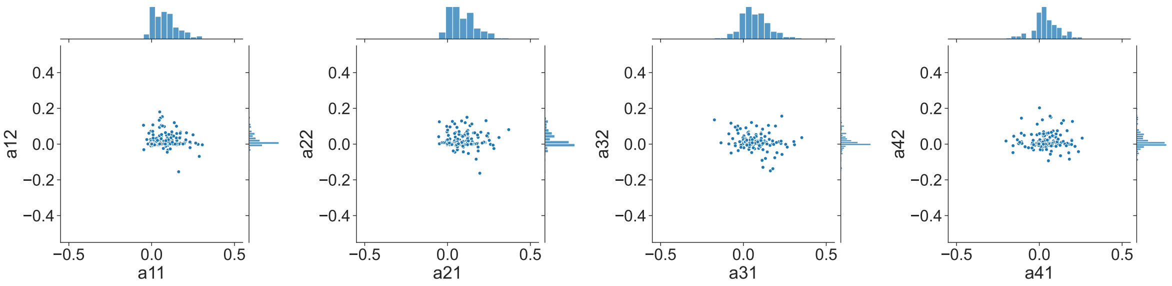

(a) (b) (c) (d)

Figure 2: The joint distributions for the MLE estimates for the eight parameters comprising the first four rows of **A** (a-d, respectively), representative of the remainder of the matrix. These were fitted with independent normal distributions.

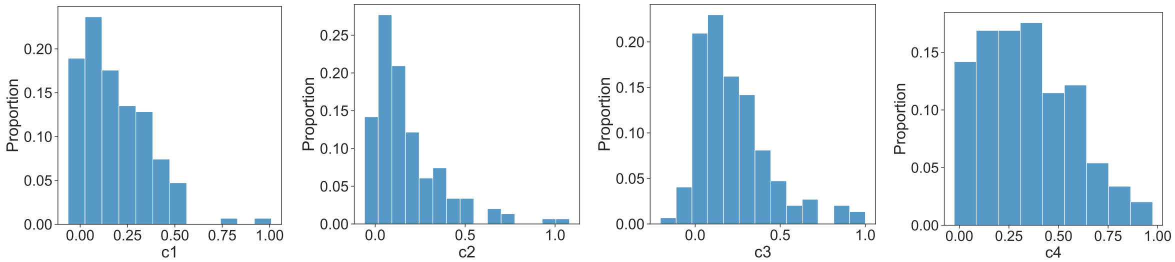

(a) (b) (c) (d)

Figure 3: Histograms of the observed distributions for the first four rows of $\boldsymbol{c}$ (a-d, respectively), representative of the remainder of the vector. These were fitted with independent normal distributions.

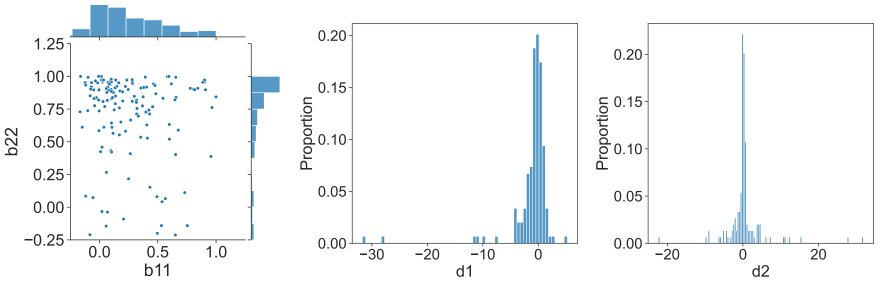

(a) (b) (c)

Figure 4: The joint distributions for the diagonal elements of $\mathbf{B}$ (a), fitted with independent truncated normal distributions, along with histograms of the two elements of $\boldsymbol{d}$ (b-c), fitted with independent normal distributions.

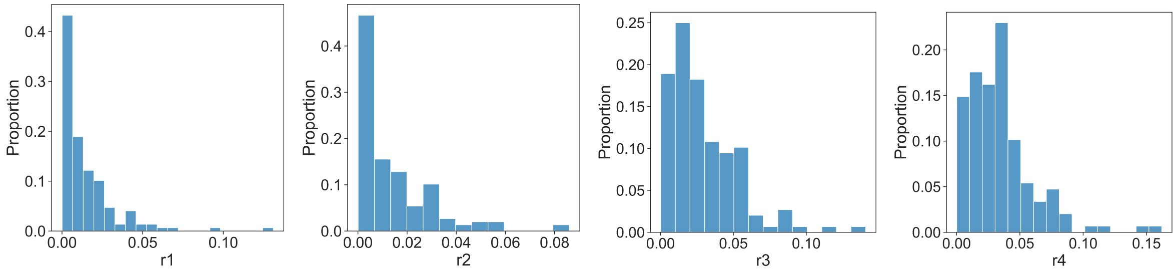

(a) (b) (c) (d)

Figure 5: Histograms of the observed distributions for the first four diagonal elements of $\mathbf{R}$ (a-d, respectively), representative of the remainder of the diagonal elements. These were fitted with independent inverse gamma distributions.

S3.7 Implementation notes

Our code implementation builds directly on that provided by the `MARSS` package [8], with Kalman filtering implemented using the `KFAS` package [7]. Fitting scripts can be found in the files `helper_functions.R` and `map_fit_driver.R` within `relevant_code/model_fitting` on OSF. Please contact the authors to discuss access to the participant data used for model training. We did not explore multiple fitting initializations as part of this project; fits were initialized using a set of parameter values provided in the project code and allowed to run for 2500 iterations with a convergence tolerance of 0.0001. Our choice of 2 hidden states

was somewhat arbitrary; 2 hidden states showed promising results when initially testing the approach using the MARSS package before creating the MAP approach described in the main manuscript. Future work will explore the impact of different numbers of latent states or latent states that correspond to interpretable quantities, such as craving. Note that we encountered some expected numerical instability for highly missing or degenerate participant data (e.g., if a participant always responded with the same value to an EMA question). We implemented a few modifications for these instances, such as updating parameters one-at-a-time and limiting the variance for EMA question observation noise to a small, but non-zero threshold.

S3.8 Modeling limitations and considerations

While our SSM framework performs well, it involves various simplifications for mathematical convenience. These simplifications allow the use of powerful existing tools, such as Kalman filtering, but should nevertheless be examined to inform future work. First, the bounded and discrete nature of EMA responses means the assumption of independent, Gaussian noise in the observation equation is flawed. An alternative would be modeling EMAs using discrete distributions, as in item-response theory. The reduction to a linear system is also a strong assumption, though this often produces good approximations in many engineering contexts. Future work will consider lapses modeled using a logit or probit transformation rather than directly modeling lapses as linear-Gaussian. Additionally, this modeling assumes a diagonal covariance matrix structure for the observation equation; given the plausible relatedness of certain questions, this is also an assumption that deserves future attention. As noted previously, this modeling uses two latent states; future work is expected to rigorously explore different latent state structures. Additionally, here we assume EMAs are missing at random, but plan to relax this assumption in future modeling. It is plausible that EMA missingness can be leveraged to gain additional insight about a patient's mental state.

There are also items worth reconsidering in the fitting approach. This work does not explore the impact of different initial parameter iterates, but we plan to study this in future work. An important assumption of the MAP fitting procedure is the structure of the parameter prior distributions, both in terms of their independence and the distributions themselves. There may be valuable covariance structures between model parameters that we currently do not model in this approach. While the chosen distributions were partially motivated to admit closed-form MAP updates, we believe they are reasonable choices for fitting the given data. In the future, we plan to explore sequential Monte Carlo (e.g., particle filtering) and Markov Chain Monte Carlo methods (e.g., using Stan) for model fitting, as they can allow for greater modeling flexibility to address the points raised above. While Monte Carlo methods are more computationally intensive, they also support more complex model definitions, such as hierarchical modeling frameworks. These approaches allow for a much richer definition of prior knowledge (as they are not limited to distributions that allow for the closed-form updates presented in this work) and multi-level pooling of information across participants. However, they will also require appropriate sensitivity analyses for the impact of different choices of prior compared to the fully data-driven prior implementation considered in this work.

S4 Machine learning models

This section provides additional details for the logistic regression (LR) and XGBoost (XGB) classifiers used as prediction benchmarks [15, 3].

S4.1 Feature construction

Both LR and XGB used a common feature set given by:

- **Day of the week:** Expressed as a one-hot-encoding for each day of the week. One of the seven booleans was dropped for logistic regression to avoid collinearity issues.
- **Most recent EMA responses:** The ordinal responses to the most recent available morning EMA scaled to [0,1]. As noted previously, the first morning EMA question dealt with reporting previous lapses, so these features correspond to the 9 ordinal responses provided from EMA questions 2-10.

- **Short-run mean of EMA responses:** The average of the participant's responses in the 3 most recent available morning EMAs (9 questions), scaled to [0,1]. Fewer EMAs were used to calculate these features when 3 EMAs were not available.
- **Long-run mean of EMA responses:** The average of the participant's responses to all available morning EMAs in their study period up to the current day (9 questions), scaled to [0,1]. For the data availability analysis, this average was calculated using smaller intervals (e.g., the most recent 15 days of data).
- **Recent lapse:** Three booleans indicating whether the individual had lapsed on any day in the last 1, 3, and 5 days, respectively.

S4.2 Hyperparameter tuning

Hyperparameters for our machine learning models were tuned by grid searching over parameter combinations (with Scikit-learn's `GridSearchCV` function). F1 score was used as the scoring mechanism for 5-fold participant stratified cross-validation to account for the class imbalance present in the data and the fact that observations are grouped by participants. Table 1 contains the parameter values used for logistic regression and Table 2 contains those used for XGBoost. The parameter grids were obtained by broader initial parameter sweeps to identify reasonable ranges for the prediction tasks evaluated in this manuscript.

Table 1: Parameters included in logistic regression parameter grid. All other parameters are set to the default values in Scikit-learn's implementation of logistic regression.

Parameter	Values
`penalty`	['l1', 'l2']
`C`	[0.001, 0.01, 0.1, 1, 10, 100, 1000]

Table 2: Parameters included in XGBoost parameter grid. All other parameters are set to the default values for the XGBoost classifier.

Parameter	Values
`n_estimators`	[25, 50, 75, 100, 125, 150, 175, 200, 225, 250, 275, 300, 325, 350, 375, 400]
`eta`	[0.1]
`colsample_bytree`	[0.75]
`subsamble`	[0.5]
`max_depth`	[1, 2, 3, 4, 5]
`min_child_weight`	[1, 2, 3, 4, 5]

S4.3 Fitting scripts

For the interested reader, the fitting scripts used for ML model training are available in the file `ml_fit.py` within `relevant_code/model_fitting` on OSF. Please contact the authors to discuss access to the participant data used for model training.

S5 Statistical analysis

The following sections describe the statistical approaches used to compare the predictive performance of the tested models.

S5.1 Overview

As noted in the manuscript, we used a Bayesian hierarchical modeling approach to evaluate model performance [1, 12]. Specifically, our analysis was implemented using the `TidyPosterior` package's `perf_mod` function [13]. This modeling uses a generalized linear model to explain the area under the receiver operating characteristic curve (auROC) performance of each model on each test fold. Using a logit link, denoted by σ, we have:

$$\sigma^{-1}(\text{auROC}_{ijk}) = b_i + c_{ij} + \beta_0 + \beta x_{ijk} + \epsilon_{ijk} \tag{56}$$

where auROC_{ijk} is the auROC performance of the kth instance of evaluation for repeat i and test fold j (here, k essentially refers to the model used). Thus the model includes a repeat specific intercept, b_i, and a fold-within-repeat specific intercept, c_{ij}. β_0 is the baseline mean model performance, β is a vector of the mean performances of each model (relative to the mean model), and x_{ijk} is a one-hot vector encoding which model was used in this k^{th} instance of repeat i and fold j. ϵ represents zero-mean Gaussian noise assumed to be common for all models. This results in the notion that auROC, in the logit transformed space, is conditionally Gaussian, i.e.:

$$\sigma^{-1}(\text{auROC}_{ijk})|b_i, c_{ij}, \beta_0, \beta, x_{ijk} \sim \mathcal{N}(b_i + c_{ij} + \beta_0 + \beta \boldsymbol{x}_{ijk}, \tilde{\sigma}^2) \tag{57}$$

where $\tilde{\sigma}^2$ is the noise variance common to all models. For the interested reader, the scripts and raw data files used for this analysis are available in `relevant_code/bayesian_hierarchical_modeling` on OSF.

S5.2 Hierarchical modeling priors

We use the default weakly-informative priors implemented by TidyPosterior, as recommended by the developers of the `rstanarm` interface to `Stan` [2]. These are as follows:

$$\begin{aligned} b_i, c_{ij}, \beta_0 &\sim \mathcal{N}(m, s_1) \\ \beta_k &\sim \mathcal{N}(0, s_2) \\ \tilde{\sigma} &\sim \exp(\lambda) \end{aligned} \tag{58}$$

Table 3 provides the parameter values for each prediction task and data availability combination.

Table 3: Prior parameters for the different prediction tasks and days of data availability, provided as (m, s_1, s_2, λ). Note that these are the default (including default adjustment), weakly informative priors recommended by `rstanarm`. Note that these prior parameters exist in the logit transformed space, not directly in the space of output auROCs.

	Prediction Task		
Data Avail.	Same-day lapse	Lapse within 3 days	Lapse within 7 days
15 days	(1.774, 0.690, 1.461, 0.276)	(1.813, 0.745, 1.577, 0.298)	(1.780, 0.782, 1.656, 0.313)
30 days	(1.848, 0.741, 1.569, 0.296)	(1.955, 0.776, 1.643, 0.310)	(1.943, 0.807, 1.708, 0.322)
45 days	(1.885, 0.813, 1.722, 0.325)	(1.987, 0.906, 1.917, 0.362)	(2.011, 1.008, 2.133, 0.403)
60 days	(2.023, 1.051, 2.225, 0.420)	(2.126, 1.247, 2.639, 0.498)	(2.202, 1.463, 3.098, 0.585)
75 days	(2.133, 1.165, 2.466, 0.466)	(2.457, 1.564, 3.311, 0.625)	(2.807, 2.419, 5.121, 0.967)

S5.3 Additional posterior reporting

Tables 4-6 provides mean and 95% credible intervals for mean model auROCs (for SSM, LR, and XGB, respectively). Table 7 provides mean and 95% credible intervals for the difference in mean model auROCs between SSM and LR (i.e., $\beta_{SSM} - \beta_{LR}$). Table 8 provides mean and 95% credible intervals for the difference in mean model auROCs between SSM and XGB (i.e., $\beta_{SSM} - \beta_{XGB}$). To make these more interpretable, mean model performance is expressed in absolute terms (not relative to the mean model parameter β_0, as is given in the original hierarchical model).

Table 4: Mean and 95% credible intervals for SSM mean model auROC for the different prediction tasks and days of data availability, provided as (`mean`, `lower`, `upper`). Credible intervals were obtained from the 2.5% and 97.5% quantiles of the posterior samples for mean model performance.

	Prediction Task		
Data Avail.	Same-day lapse	Lapse within 3 days	Lapse within 7 days
15 days	(0.862, 0.854, 0.869)	(0.848, 0.839, 0.857)	(0.837, 0.827, 0.847)
30 days	(0.876, 0.869, 0.883)	(0.886, 0.879, 0.893)	(0.880, 0.871, 0.888)
45 days	(0.876, 0.867, 0.884)	(0.883, 0.874, 0.892)	(0.885, 0.875, 0.895)
60 days	(0.897, 0.888, 0.906)	(0.908, 0.898, 0.917)	(0.911, 0.900, 0.922)
75 days	(0.906, 0.897, 0.915)	(0.934, 0.925, 0.942)	(0.956, 0.946, 0.964)

Table 5: Mean and 95% credible intervals for LR mean model auROC for the different prediction tasks and days of data availability, provided as (`mean`, `lower`, `upper`). Credible intervals were obtained from the 2.5% and 97.5% quantiles of the posterior samples for mean model performance.

	Prediction Task		
Data Avail.	Same-day lapse	Lapse within 3 days	Lapse within 7 days
15 days	(0.860, 0.853, 0.868)	(0.864, 0.855, 0.872)	(0.865, 0.856, 0.873)
30 days	(0.867, 0.859, 0.874)	(0.868, 0.860, 0.876)	(0.871, 0.862, 0.879)
45 days	(0.874, 0.865, 0.885)	(0.877, 0.868, 0.886)	(0.880, 0.870, 0.890)
60 days	(0.885, 0.875, 0.895)	(0.884, 0.873, 0.896)	(0.891, 0.877, 0.904)
75 days	(0.897, 0.887, 0.907)	(0.915, 0.903, 0.925)	(0.939, 0.925, 0.951)

Table 6: Mean and 95% credible intervals for XGB mean model auROC for the different prediction tasks and days of data availability, provided as (`mean`, `lower`, `upper`). Credible intervals were obtained from the 2.5% and 97.5% quantiles of the posterior samples for mean model performance.

	Prediction Task		
Data Avail.	Same-day lapse	Lapse within 3 days	Lapse within 7 days
15 days	(0.840, 0.831, 0.848)	(0.866, 0.858, 0.874)	(0.862, 0.853, 0.871)
30 days	(0.847, 0.838, 0.855)	(0.872, 0.864, 0.880)	(0.871, 0.863, 0.880)
45 days	(0.853, 0.843, 0.862)	(0.877, 0.867, 0.886)	(0.879, 0.869, 0.889)
60 days	(0.862, 0.851, 0.874)	(0.885, 0.873, 0.896)	(0.897, 0.884, 0.909)
75 days	(0.875, 0.863, 0.886)	(0.911, 0.899, 0.922)	(0.929, 0.913, 0.943)

Table 7: Posterior probability that the mean model auROC for SSM is larger than LR, as well as mean and 95% credible intervals for the raw difference in mean model auROC between SSM and LR for the different prediction tasks and days of data availability, provided as (`probability`, `mean`, `lower`, `upper`). Credible intervals were obtained from the 2.5% and 97.5% quantiles of the posterior samples for mean model performance.

	Prediction Task		
Data Avail.	Same-day lapse	Lapse within 3 days	Lapse within 7 days
15 days	(0.732, 0.001, -0.003, 0.006)	(<0.001, -0.015, -0.020, -0.011)	(<0.001, 0.027, -0.033, -0.021)
30 days	(0.999, 0.009, 0.004, 0.014)	(0.999, 0.017, 0.013, 0.021)	(0.999, 0.009, 0.003, 0.014)
45 days	(0.760, 0.002, -0.003, 0.007)	(0.992, 0.006, 0.001, 0.011)	(0.939, 0.005, -0.001, 0.011)
60 days	(0.999, 0.012, 0.006, 0.018)	(0.999, 0.023, 0.018, 0.029)	(0.999, 0.020, 0.012, 0.028)
75 days	(0.995, 0.009, 0.002, 0.016)	(0.999, 0.019, 0.011, 0.027)	(0.999, 0.016, 0.006, 0.027)

Table 8: Posterior probability that the mean model auROC for SSM is larger than XGB, as well as mean and 95% credible intervals for the raw difference in mean model auROC between SSM and XGB for the different prediction tasks and days of data availability, provided as (`probability`, `mean`, `lower`, `upper`). Credible intervals were obtained from the 2.5% and 97.5% quantiles of the posterior samples for mean model performance.

	Prediction Task		
Data Avail.	Same-day lapse	Lapse within 3 days	Lapse within 7 days
15 days	(0.999, 0.022, 0.016, 0.027)	(<0.001, -0.017, -0.022, -0.013)	(<0.001, -0.024, -0.030, -0.018)
30 days	(0.999, 0.029, 0.023, 0.034)	(0.999, 0.014, 0.010, 0.017)	(0.998, 0.008, 0.003, 0.013)
45 days	(0.999, 0.023, 0.016, 0.029)	(0.991, 0.006, 0.001, 0.011)	(0.962, 0.005, -0.001, 0.012)
60 days	(0.999, 0.034, 0.028, 0.041)	(0.999, 0.023, 0.017, 0.029)	(0.999, 0.014, 0.007, 0.022)
75 days	(0.999, 0.031, 0.023, 0.039)	(0.999, 0.023, 0.015, 0.032)	(0.999, 0.026, 0.014, 0.039)

S6 Additional plots

This section includes additional plots referenced in the main manuscript.

S6.1 Raw auROC values

Figure 6 provides the raw auROC values obtained for each combination of prediction task and data availability from 15 repeats of 5-fold cross validation. Note that we observe much greater variance than in the posterior samples for mean model performance. This is expected, as these raw auROC values contain the variance related to, for example, varying difficulty across test folds in the cross-validation process.

S6.2 Posterior mean auROC correlation

The purpose of the Bayesian hierarchical analysis is to quantify our uncertainty in different parts of the model via the joint posterior distribution of parameters in the model. Given the specified priors and the observed data, TidyPosterior uses Hamiltonian Monte Carlo methods (via `Stan` [2]) to generate samples approximating this posterior joint distribution. We then use these posterior samples to generate useful summaries of the posterior distribution. Note that each posterior sample contains a value for all model parameters. For example, we can generate credible intervals for a model's mean auROC by looking only at that model's auROC parameter and examining the corresponding distribution across all samples. However, it is important to note that there can be correlations between parameters in the joint posterior. For instance, consider the same-day prediction credible intervals provided in Figure 5 of the main manuscript, specifically at 15 and 30 days of data availability. For these cases, SSM and LR show the best performance, but have

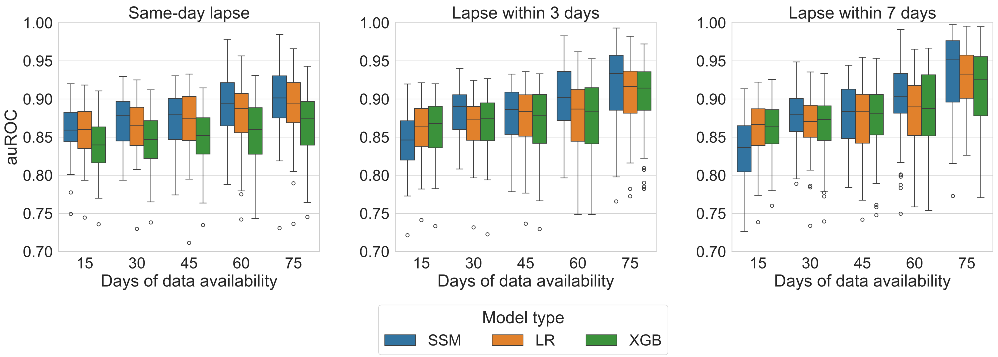

Figure 6: Box plots of auROC performance from repeats of the participant stratified 5-fold cross validation process. Medians are denoted with horizontal lines, boxes denote the interquartile range, whiskers denote non-outlier minima and maxima, and dots denote outliers. Each pane describes performance on a different prediction task, ranging from same-day prediction to week-long window prediction. Note that these are the raw auROC values from each iteration of cross-validation, which have greater uncertainties than the mean model auROCs assessed as part of the hierarchical modeling.

considerable overlap in their mean auROC credible intervals. Despite this, the posterior probabilities (see Table 2 in the main manuscript) that SSM is the best performing method are 0.732 and 0.999 respectively. Figure 7 shows joint scatterplots displaying samples of mean auROC performance for SSM and LR in these two prediction contexts.

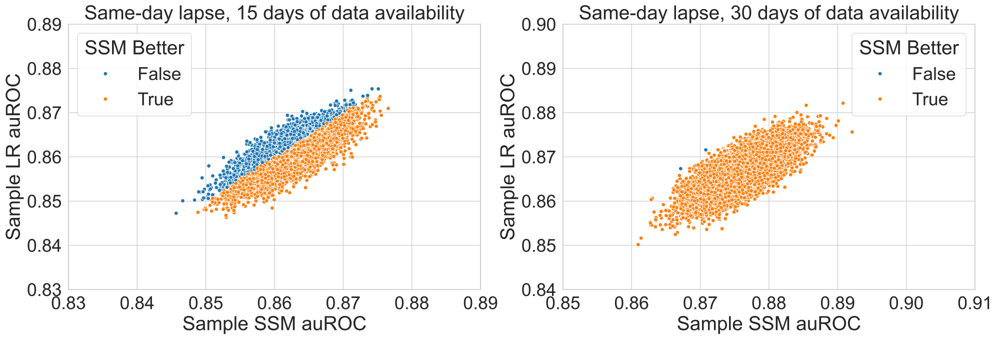

Figure 7: Scatterplots of posterior samples, plotting the value of the mean SSM auROC parameter on the x-axis and the value of the mean LR auROC parameter on the y-axis. The left pane shows samples from the case where 15 days of data availability are available and the right pane shows samples for the case where 30 days of data availability are available (both refer to same-day prediction). Dots are colored based on whether the SSM auROC value is higher than the LR auROC value. The relative fraction of each color give insight into the calculated posterior probabilities that SSM has the highest mean auROC performance (0.732 and 0.999, respectively).

While there is uncertainty in the mean model auROC of both models, there is far less uncertainty in the relative ranking of these two models. As a result, summarizing credible intervals for separate parameters (e.g., the mean auROC performance of each model) and comparing these credible intervals offers an incomplete view for ranking relative model performance. Hence, in the main manuscript, we evaluate the posterior probability that the SSMs had the best mean auROC performance. This was done by evaluating, for each sample, if the mean auROC value associated with the SSM was higher than the corresponding values for both LR and XGB and then taking the average of this binary value across all samples. This approach thus properly considers the correlated uncertainty in model performance, but allows the reader to draw a straightforward conclusion regarding our posterior uncertainty in model ranking.

S6.3 Area under the precision-recall curve

For completeness, we also provide analogous plots of the area under the precision-recall curve (auPRC). Figure 8 provides the raw auPRC values from repeated cross-fold validation. Figure 9 provides the mean auPRC values obtained by the Bayesian hierarchical modeling for auPRC (using exactly the same processing approach, but replacing auROC values with auPRC values). Table 9 provides posterior probabilities that the SSMs have the best mean auPRC of the tested methods.

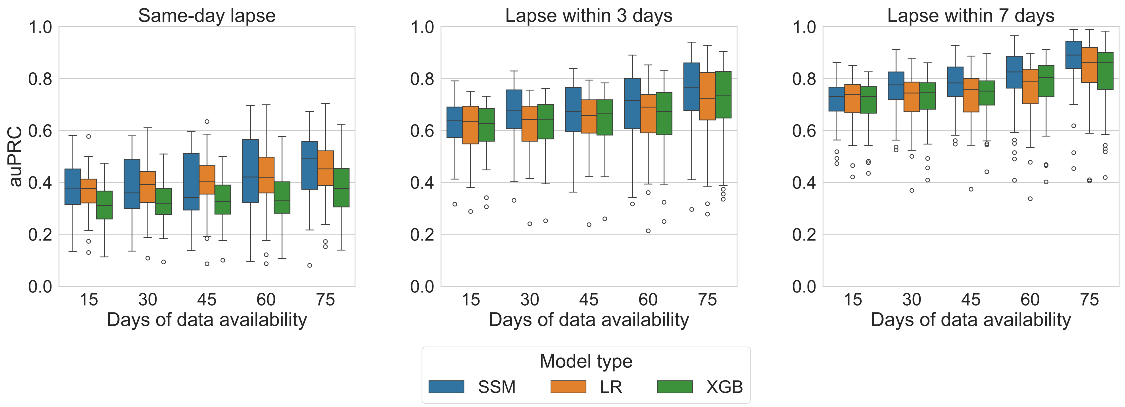

Figure 8: Box plots of mean auPRC performance from repeats of the participant stratified 5-fold cross validation process. Medians are denoted with horizontal lines, boxes denote the interquartile range, whiskers denote non-outlier minima and maxima, and dots denote outliers. Each pane describes performance on a different prediction task, ranging from same-day prediction to week-long window prediction. Note that these are the raw auPRC values from each iteration of cross-validation, which have greater uncertainties than the mean model auPRCs assessed as part of the hierarchical modeling.

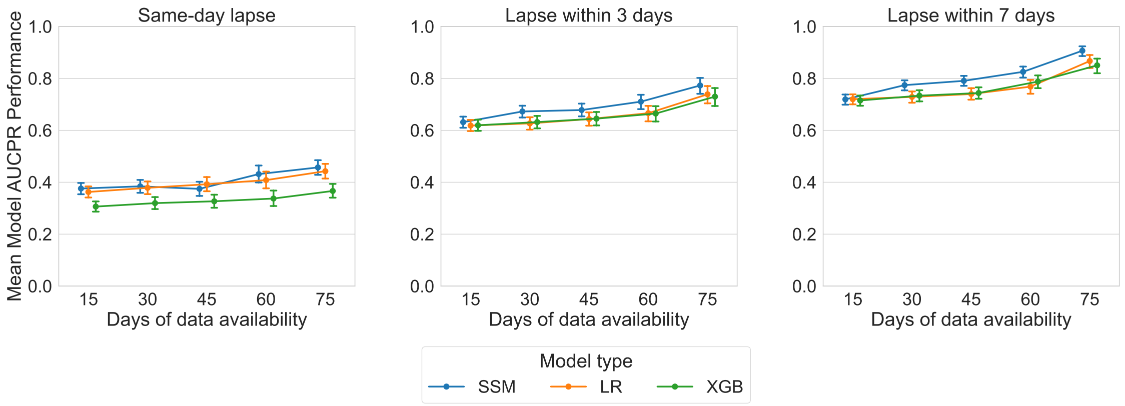

Figure 9: Plots of posterior samples of mean auPRC performance for the three methods. Medians are marked with dots and 95% credible intervals (CrI) are given as error bars. Each panel describes performance on a different prediction task. Plotted values for each number of days of data availability are offset slightly for easier viewing of the median and CrI bars. Similar to auROC, there is correlation between the models in the posterior, so the clearest summary of model performance ranking is provided by the posterior summaries in Table 9.

Table 9: Each cell contains the posterior probability that the SSM model has the best mean auPRC performance of the three methods used on this dataset. These probabilities were calculated by drawing posterior samples of model performance from the Bayesian hierarchical model and identifying the fraction of samples with mean SSM auPRC performance greater than that of LR and XGB.

	Data availability (days)				
Prediction Task	15	30	45	60	75
Same-day lapse	0.954	0.754	0.024	0.984	0.917
Lapse within 3 days	0.985	0.999	0.999	0.999	0.999
Lapse within 7 days	0.395	0.999	0.999	0.999	0.999

References

[1] Alessio Benavoli et al. "Time for a Change: A Tutorial for Comparing Multiple Classifiers through Bayesian Analysis". In: *Journal of Machine Learning Research* 18.77 (2017), pp. 1–36. URL: http://jmlr.org/papers/v18/16-305.html.

[2] Bob Carpenter et al. "*Stan* : A Probabilistic Programming Language". In: *Journal of Statistical Software* 76.1 (2017). ISSN: 1548-7660. DOI: 10.18637/jss.v076.i01. URL: http://www.jstatsoft.org/v76/i01/.

[3] Tianqi Chen and Carlos Guestrin. "XGBoost: A Scalable Tree Boosting System". In: *Proceedings of the 22nd ACM SIGKDD International Conference on Knowledge Discovery and Data Mining*. KDD '16: The 22nd ACM SIGKDD International Conference on Knowledge Discovery and Data Mining. San Francisco California USA: ACM, Aug. 13, 2016, pp. 785–794. ISBN: 978-1-4503-4232-2. DOI: 10.1145/2939672.2939785. URL: https://dl.acm.org/doi/10.1145/2939672.2939785.

[4] Marie Laure Delignette-Muller and Christophe Dutang. "fitdistrplus: An R Package for Fitting Distributions". In: *Journal of Statistical Software* 64.4 (2015), pp. 1–34. DOI: 10.18637/jss.v064.i04.

[5] A. P. Dempster, N. M. Laird, and D. B. Rubin. "Maximum Likelihood from Incomplete Data via the EM Algorithm". In: *Journal of the Royal Statistical Society. Series B (Methodological)* 39.1 (1977), pp. 1–38. ISSN: 0035-9246. JSTOR: 2984875. URL: https://www.jstor.org/stable/2984875.

[6] J. Durbin and S. J. Koopman. *Time Series Analysis by State Space Methods*. Oxford Statistical Science Series 24. Oxford ; New York: Oxford University Press, 2001. 253 pp. ISBN: 978-0-19-852354-3.

[7] Jouni Helske. "**KFAS** : Exponential Family State Space Models in *R*". In: *Journal of Statistical Software* 78.10 (2017). ISSN: 1548-7660. DOI: 10.18637/jss.v078.i10. URL: http://www.jstatsoft.org/v78/i10/.

[8] Elizabeth Holmes E., Eric Ward J., and Kellie Wills. "MARSS: Multivariate Autoregressive State-space Models for Analyzing Time-series Data". In: *The R Journal* 4.1 (2012), p. 11. ISSN: 2073-4859. DOI: 10.32614/RJ-2012-002. URL: https://journal.r-project.org/archive/2012/RJ-2012-002/index.html.

[9] Elizabeth E. Holmes. *Derivation of an EM Algorithm for Constrained and Unconstrained Multivariate Autoregressive State-Space (MARSS) Models*. Feb. 15, 2013. arXiv: 1302.3919 [stat]. URL: http://arxiv.org/abs/1302.3919. Pre-published.

[10] Roger A. Horn and Charles R. Johnson. *Topics in Matrix Analysis*. Cambridge ; New York: Cambridge University Press, 1991. 607 pp. ISBN: 978-0-521-30587-7.

[11] R. E. Kalman. "A New Approach to Linear Filtering and Prediction Problems". In: *Journal of Basic Engineering* 82.1 (Mar. 1, 1960), pp. 35–45. ISSN: 0021-9223. DOI: 10.1115/1.3662552. URL: https://asmedigitalcollection.asme.org/fluidsengineering/article/82/1/35/397706/A-New-Approach-to-Linear-Filtering-and-Prediction.

[12] John K. Kruschke and Torrin M. Liddell. "The Bayesian New Statistics: Hypothesis Testing, Estimation, Meta-Analysis, and Power Analysis from a Bayesian Perspective". In: *Psychonomic Bulletin & Review* 25.1 (Feb. 2018), pp. 178–206. ISSN: 1069-9384, 1531-5320. DOI: 10.3758/s13423-016-1221-4. URL: http://link.springer.com/10.3758/s13423-016-1221-4.

[13] Max Kuhn. *Tidyposterior: Bayesian Analysis to Compare Models Using Resampling Statistics.* manual. 2023.

[14] Andrew Ng and Tengyu Ma. *Machine Learning.* CS229 Lecture Notes. 2022. URL: https://cs229.stanford.edu/lectures-spring2022/main_notes.pdf.

[15] Fabian Pedregosa et al. "Scikit-Learn: Machine Learning in Python". In: *Journal of Machine Learning Research* 12.85 (2011), pp. 2825–2830. ISSN: 1533-7928. URL: http://jmlr.org/papers/v12/pedregosa11a.html.

[16] Kaare Brandt Petersen and Michael Syskind Pedersen. *The Matrix Cookbook.* 2008.

[17] H. E. Rauch, F. Tung, and C. T. Striebel. "Maximum Likelihood Estimates of Linear Dynamic Systems". In: *AIAA Journal* 3.8 (Aug. 1965), pp. 1445–1450. ISSN: 0001-1452, 1533-385X. DOI: 10.2514/3.3166. URL: https://arc.aiaa.org/doi/10.2514/3.3166.

[18] Sam Roweis and Zoubin Ghahramani. "A Unifying Review of Linear Gaussian Models". In: *Neural Computation* 11.2 (Feb. 1, 1999), pp. 305–345. ISSN: 0899-7667, 1530-888X. DOI: 10.1162/089976699300016674. URL: https://direct.mit.edu/neco/article/11/2/305-345/6249.

[19] Robert H. Shumway and David S. Stoffer. *Time Series Analysis and Its Applications: With R Examples.* 4th ed. 2017. Springer Texts in Statistics. Cham: Springer International Publishing : Imprint: Springer, 2017. 1 p. ISBN: 978-3-319-52452-8. DOI: 10.1007/978-3-319-52452-8.

[20] W. N. Venables and B. D. Ripley. *Modern Applied Statistics with S.* 4th ed. New York: Springer, 2002. URL: https://www.stats.ox.ac.uk/pub/MASS4/.

[21] Kendra Wyant et al. "Machine Learning Models for Temporally Precise Lapse Prediction in Alcohol Use Disorder." In: *Journal of Psychopathology and Clinical Science* (Aug. 22, 2024). ISSN: 2769-755X, 2769-7541. DOI: 10.1037/abn0000901. URL: https://doi.apa.org/doi/10.1037/abn0000901.
