# Supplementary material for: Idiographic Lapse Prediction With State Space Modeling: Algorithm Development and Validation Study
Source: JMIR Form Res. 2025 Jun 3;9:e73265. doi: 10.2196/73265 (PMC12174888; doi:10.2196/73265)
Supplement: Multimedia Appendix 2 [file formative_v9i1e73265_app2.docx]

Transparency Report 1.0 (full, 36 items) (Aczel et al. 2019)

**Manuscript Title:** Idiographic Lapse Prediction with State Space Modeling: Algorithm Development and Validation

**Authors**: Eric Pulick, John J. Curtin, & Yonatan Mintz

**Corresponding author’s email address**: pulick@wisc.edu

**Link to Project Repository**: https://osf.io/8xbk2/

**Preregistration Section**

• Prior to analyzing the complete data set, a time-stamped preregistration was posted in an independent, third-party registry for the data analysis plan: Yes. see https://osf.io/8xbk2/registrations

**Methods Section**

The manuscript fully describes…

**• the rationale for the sample size used (e.g., an a priori power analysis)**: Yes

**• how participants were recruited**: Yes

**• how participants were selected (e.g., eligibility criteria)**: Yes

**• what compensation was offered for participation**: Yes

**• how participant dropout was handled (e.g., replaced, omitted, etc)**: Yes

**• how participants were assigned to conditions**: N/A. There are no conditions.

**• how stimulus materials were randomized**: N/A.

**• whether (and, if so, how) participants, experimenters, and data-analysts were kept naive to potentially biasing information**: N/A. This is an observations study that does not include analysis of group or manipulations. There were no study conditions to blind.

**• the study design, procedures, and materials to allow independent replication:** Yes

**• the measures of interest (e.g., friendliness)**: Yes

**• all operationalizations for the measures of interest (e.g., a questionnaire measuring friendliness)**: Yes

**Results and Discussion Section**

The manuscript…

• **distinguishes explicitly between “confirmatory” (i.e., prespecified) and “exploratory” (i.e., not prespecified) analyses**: All reported analyses were pre-specified.

• **describes how violations of statistical assumptions were handled**: Yes, we used a logit transformation for auROC and specify this in the manuscript.

**• justifies all statistical choices (e.g., including or excluding covariates; applying or not applying transformations; use of multi-level models vs. ANOVA)**: Yes

**• reports the sample size for each cell of the design**: Yes

**• reports how incomplete or missing data were handled**: Yes

**• presents protocols for data preprocessing (e.g., cleaning, discarding of cases and items, normalizing, smoothing, artifact correction)**: Yes

**Data, Code, and Materials Availability Section**

The following have been made publicly available…

**• the (processed) data, on which the analyses of the manuscript were based**: Yes, see OSF

**• all code and software (that is not copyright protected)**: Yes, see OSF

**• all instructions, stimuli, and test materials (that are not copyright protected)**: Yes, see OSF

**• Are the data properly archived (i.e., would a graduate student with relevant background knowledge be able to identify each variable and reproduce the analysis)**: Yes

**• The manuscript includes a statement concerning the availability and location of all research items, including data, materials, and code relevant to the study**: Yes

**References**

Aczel B, Szaszi B, Sarafoglou A, Kekecs Z, Kucharský Š, Benjamin D, Chambers CD, Fisher A, Gelman A, Gernsbacher MA, Ioannidis JP, Johnson E, Jonas K, Kousta S, Lilienfeld SO, Lindsay DS, Morey CC, Munafò M, Newell BR, Pashler H, Shanks DR, Simons DJ, Wicherts JM, Albarracin D, Anderson ND, Antonakis J, Arkes HR, Back MD, Banks GC, Beevers C, Bennett AA, Bleidorn W, Boyer TW, Cacciari C, Carter AS, Cesario J, Clifton C, Conroy RM, Cortese M, Cosci F, Cowan N, Crawford J, Crone EA, Curtin J, Engle R, Farrell S, Fearon P, Fichman M, Frankenhuis W, Freund AM, Gaskell MG, Giner-Sorolla R, Green DP, Greene RL, Harlow LL, de la Guardia FH, Isaacowitz D, Kolodner J, Lieberman D, Logan GD, Mendes WB, Moersdorf L, Nyhan B, Pollack J, Sullivan C, Vazire S, Wagenmakers E. A consensus-based transparency checklist. Nat Hum Behav 2019; 4(1):4-6
