# Supplementary material for: Idiographic Lapse Prediction With State Space Modeling: Algorithm Development and Validation Study
Source: JMIR Form Res. 2025 Jun 3;9:e73265. doi: 10.2196/73265 (PMC12174888; doi:10.2196/73265)
Supplement: Multimedia Appendix 3 [file formative_v9i1e73265_app3.pdf]

Consolidated reporting guidelines for prognostic and diagnostic machine learning modeling studies

Multimedia Appendix 3: Author Checklist

The following is the reporting checklist. A response should indicate whether the particular item is documented in the study. If the response to an item is Y then the location in the article should be provided (e.g., section number), and if the response is N or NA then some reasoning should be provided.

#	Item	Y	N	NA	Location / Reasoning
**Study Details**					
1.1	*The medical/clinical task of interest*	X			'Introduction' and 'Labels and prediction tasks'
1.2	*The research question*	X			'Introduction'
1.3	*Current medical/clinical practice*	X			'Introduction'
1.4	*The known predictors and confounders to what is being predicted / diagnosed*	X			'Introduction,' 'Measures,' and 'Labels and prediction tasks'
1.5	*The overall study design*	X			'Ethical considerations' and 'Recruitment'
1.6	*The medical institutional setting(s)*	X			'Recruitment' and 'Measures'
1.7	*The target patient population*	X			'Recruitment'
1.8	*The intended use of the ML model*	X			'Statistical analysis of model comparisons' and 'Extensions to intervention frameworks'
1.9	*Existing model performance benchmarks for this task*	X			'Introduction' and 'Lapse prediction performance'
1.10	*Ethical and other regulatory approvals obtained*	X			'Ethical considerations' and 'Transparency and openness'
**The Data**					
2.1	*Inclusion / exclusion criteria for the patient cohort*	X			'Measures'
2.2	*Methods of data collection*	X			'Recruitment' and 'Measures'
2.3	*Bias introduced due to the method of data collection used*	X			'Actively collected patient data have

					important limitations and potential biases’
2.4	*Data characteristics*	X			‘Results’ (the subsections describe various characteristics of the data)
2.5	*Methods of data transformations and preprocessing applied*	X			‘Measures,’ ‘Labels and prediction tasks,’ ‘State space modeling,’ and ‘Benchmark machine learning methods’
2.6	*Known quality issues with the data*	X			‘Actively collected patient data have important limitations and potential biases’
2.7	*Sample size calculation*	X			‘Recruitment’
2.8	*Data Availability*	X			‘Data availability’
**Methodology**					
3.1	*Strategies for handling missing data*	X			‘State space modeling’ and ‘Benchmark machine learning methods’
3.2	*Strategies for addressing class imbalance*	X			‘Statistical analysis of model comparisons’
3.3	*Strategies for reducing dimensionality of data*			X	This is not a concern for this dataset, though we do discuss some tangential aspects of how the state space models perform dimensionality reduction through their definition of latent states in ‘Limited interpretability of these SSMs due to latent state definition’
3.4	*Strategies for handling outliers*			X	The bounded nature of EMA responses and lapse values are such that outliers are not a concern in the

					measured data. The only remaining outlier handling was in our exclusion protocol (addressed by 2.1), where we chose to exclude 3 participants due to exceedingly low EMA compliance.
3.5	*Strategies for data augmentation*			X	Data were not augmented as part of this analysis.
3.6	*Strategies for model pre-training*	X			'Fitting procedures'
3.7	*The rationale for selecting the machine learning algorithm*	X			'Introduction,' 'State space modeling,' and 'Benchmark machine learning methods'
3.8	*The method of evaluating model performance during training*	X			'Fitting procedures' and 'Statistical analysis of model comparisons'
3.9	*The method used for hyperparameter tuning*	X			'Fitting procedures'
3.10	*Model's output adjustments*	X			'Statistical analysis of model comparisons'
**Evaluation**					
4.1	*Performance metrics used to evaluate the model*	X			'Statistical analysis of model comparisons'
4.2	*The cost or consequence of errors*	X			'Statistical analysis of model comparisons'
4.3	*The results of internal validation*	X			'Results' and Sections E-F of Multimedia Appendix 1
4.4	*The final model hyperparameters*	X			Section D of Multimedia Appendix 1
4.5	*Model evaluation on an external dataset*			X	Our model was trained and evaluated on a single cohort using repeated cross-validation (see 'Fitting procedures')

4.6	*Characteristics relevant for detecting data shift and drift*	X			'Limited demographic diversity in study population' as well as a discussion of the importance of model personalization in 'Introduction' and 'State space modeling'
**Explainability and Transparency**					
5.1	*The most important features and how they relate to the outcome(s)*	X			'Principal findings'
5.2	*Plausibility of model outputs*	X			'Principal findings' (i.e., comparison to benchmark methods from the literature)
5.3	*Interpretation of model's results by an end-user*	X			'Clinical interpretability of modeling approaches'
